# Predicting delivery of a small‐for‐gestational‐age infant and adverse perinatal outcome in women with suspected pre‐eclampsia

**DOI:** 10.1002/uog.17490

**Published:** 2018-02-07

**Authors:** M. Griffin, P. T. Seed, S. Duckworth, R. North, J. Myers, L. Mackillop, N. Simpson, J. Waugh, D. Anumba, L. C. Kenny, C. W. G. Redman, A. H. Shennan, L. C. Chappell

**Affiliations:** ^1^ Women's Health Academic Centre King's College London London UK; ^2^ Maternal and Fetal Health Research Centre University of Manchester Manchester UK; ^3^ Oxford University Hospital NHS Trust Oxford UK; ^4^ Division of Women's and Children's Health, Faculty of Health University of Leeds Leeds UK; ^5^ Newcastle upon Tyne Hospitals NHS Foundation Trust Newcastle UK; ^6^ Academic Unit of Reproductive and Developmental Medicine University of Sheffield Sheffield UK; ^7^ INFANT Irish Centre for Fetal and Neonatal Translational Research University College Cork Cork Ireland; ^8^ Nuffield Department of Obstetrics and Gynaecology University of Oxford Oxford UK

**Keywords:** growth restriction, placental growth factor, pre‐eclampsia, small‐for‐gestational‐age

## Abstract

**Objective:**

To evaluate the test performance of 47 biomarkers and ultrasound parameters for the prediction of delivery of a small‐for‐gestational‐age (SGA) infant and adverse perinatal outcome in women presenting with suspected pre‐eclampsia.

**Methods:**

This was a prospective, multicenter observational study in which 47 biomarkers and ultrasound parameters were measured in 397 women with a singleton pregnancy presenting with suspected preterm pre‐eclampsia between 20 + 0 and 36 + 6 weeks' gestation, with the objective of evaluating them as predictors of subsequent delivery of a SGA infant and adverse perinatal outcome. Women with confirmed pre‐eclampsia at enrollment were excluded. Factor analysis and stepwise logistic regression were performed in two prespecified groups stratified according to gestational age at enrollment. The primary outcome was delivery of a SGA infant with a birth weight < 3^rd^ customized centile (SGA‐3), and secondary outcomes were a SGA infant with a birth weight < 10^th^ customized centile and adverse perinatal outcome.

**Results:**

In 274 women presenting at 20 + 0 to 34 + 6 weeks' gestation, 96 (35%) delivered a SGA‐3 infant. For prediction of SGA‐3, low maternal placental growth factor (PlGF) concentration had a sensitivity of 93% (95% CI, 84–98%) and negative predictive value (NPV) of 90% (95% CI, 76–97%) compared with a sensitivity of 71% (95% CI, 58–82%) and a NPV of 79% (95% CI, 68–87%) for ultrasound parameters (estimated fetal weight or abdominal circumference < 10^th^ centile). No individual biomarker evaluated had a better performance than did PlGF, and marker combinations made only small improvements to the test performance. Similar results were found in 123 women presenting between 35 + 0 and 36 + 6 weeks' gestation.

**Conclusion:**

In women presenting with suspected preterm pre‐eclampsia, measurement of PlGF offers a useful adjunct for identifying those at high risk of delivering a SGA infant, allowing appropriate surveillance and timely intervention. © 2017 The Authors. *Ultrasound in Obstetrics & Gynecology* published by John Wiley & Sons Ltd on behalf of the International Society of Ultrasound in Obstetrics and Gynecology.

## Abstract 2

Linked Comment: Ultrasound Obstet Gynecol 2018; 51: 304–305

## INTRODUCTION

Infants who are born small‐for‐gestational age (SGA) are at increased risk of short‐term neonatal morbidity and mortality^1^, ^2^, ^3^, and longer‐term complications extending into adult life, including cardiovascular disease and Type‐II diabetes mellitus^4^. SGA is commonly defined as birth weight under a centile threshold. For infants under the 10^th^ centile of the population, this group includes constitutionally small infants and those with fetal growth restriction, the latter defined as failure of a fetus to reach its full growth potential. Use of birth‐weight centiles customized for additional maternal (height, weight, ethnicity, parity) and fetal (sex) variables improves identification of those fetuses at risk of adverse perinatal outcome, including stillbirth and neonatal death^5^.

The underlying pathophysiology of fetal growth restriction is complex, but poor placentation plays a key role in a substantial proportion of SGA, particularly in women with preterm hypertensive disorders and when associated with adverse perinatal outcome. There is a need for a test in the second half of pregnancy to identify those at highest risk of delivering a SGA infant. Markers of placental function could offer a useful adjunct to current ultrasonographic techniques to improve risk stratification, enabling identification of those at greatest risk and minimizing unnecessary interventions in lower‐risk women. Several biomarkers have been suggested as potential predictors of fetal growth restriction, but to date none has been shown to have adequate accuracy to support incorporation into clinical practice^6^. The fetuses of women with suspected hypertensive disorders of pregnancy who present before 37 weeks' gestation are at increased risk of fetal growth restriction, but the optimal strategy for identifying such fetuses remains unclear.

As part of a large prospective study in women presenting with suspected pre‐eclampsia (PE), we sought first to evaluate 47 biomarkers (identified by an extensive literature search) and then compare the best performing biomarker(s) against currently utilized ultrasound parameters for determining subsequent delivery of a SGA infant and adverse perinatal outcome.

## METHODS

The PELICAN study was a prospective observational study, undertaken between January 2011 and February 2012 in seven consultant‐led maternity units in the UK and Ireland. The role of placental growth factor (PlGF) in determining the need for delivery within 14 days of sampling for PE in this study has been reported previously^7^, and this was a planned further analysis.

### Participants

Study eligibility required the presence of signs or symptoms of suspected PE in women presenting between 20 + 0 and 36 + 6 weeks' gestation with a singleton pregnancy and aged ≥ 16 years; women with confirmed PE at enrollment were excluded. Written informed consent was obtained and baseline demographic and pregnancy‐specific data were entered into the study database. Blood was drawn into ethylenediamine‐tetraacetic acid at study enrollment and samples were centrifuged at 3000 rpm for 10 min. Plasma was extracted and stored at –80°C until analysis. Management of the women in the study followed the usual care pathways for women with suspected PE, as advised in the UK National Institute for Health and Care Excellence ‘Hypertension in Pregnancy’ guidelines^8^, with ultrasound assessment being undertaken as clinically indicated.

Ultrasound assessments were undertaken by trained ultrasonographers at each study site as clinically indicated, using a variety of machines and following local protocols for the measurement of fetal biometry, amniotic fluid index and umbilical artery Doppler flow‐velocity waveforms (as occurred in clinical practice at the time of the study). Quality control was undertaken through local procedures rather than by the research team centrally. Estimated fetal weight (EFW) was calculated at each site using the Hadlock formula^9^. Additional parameters, including uterine artery, fetal middle cerebral artery and ductus venosus Doppler studies, were not universally reported and therefore their performance could not be compared with biomarker performance. As study sites were reporting abnormal ultrasound assessment using a variety of parameters (including abdominal circumference (AC) and EFW < 10^th^, < 5^th^ and < 3^rd^ centiles), the most commonly reported parameter, AC or EFW < 10^th^ centile, was chosen to enable comparison across sites. The presence of AC or EFW < 10^th^ centile, oligohydramnios (amniotic fluid index < 5^th^ centile) or absent/reversed end‐diastolic flow was recorded by participating midwives.

Final diagnoses of maternal hypertensive disorders of pregnancy were assigned, following agreement by an adjudication panel of experts, using definitions from the American College of Obstetricians and Gynecologists practice bulletin^10^. SGA was defined as birth weight < 3^rd^ customized centile (SGA‐3), with birth weight < 10^th^ customized centile (SGA‐10) being a secondary outcome, calculated using the Gestation‐Related Optimal Weight (GROW) method by freely available software^11^. All diagnoses were assigned without knowledge of any biomarker values.

The prespecified first part of the biomarker analysis presented here relates to two groups of women in predefined gestational age strata enrolled with a singleton pregnancy and suspected preterm PE: Group 1 at 20 + 0 to 34 + 6 weeks' gestation and Group 2 at 35 + 0 to 36 + 6 weeks' gestation. For comparison with ultrasound parameters, the second part of the analysis was restricted to women with an ultrasound scan performed within 14 days of blood sampling at enrollment. The principal prespecified outcome of both analyses was delivery of a SGA‐3 infant^3^. The prespecified secondary outcome measures were birth weight < 10^th^ customized centile (SGA‐10) and adverse perinatal outcome. Adverse perinatal outcome was predefined as the presence of any of the following complications: antepartum/intrapartum fetal or neonatal death, intraventricular hemorrhage, periventricular leukomalacia, seizure, retinopathy of prematurity, respiratory distress syndrome, bronchopulmonary dysplasia, necrotizing enterocolitis or admission to neonatal unit for > 48 h at term. Adverse maternal outcome was defined as the presence of any of the following: maternal death, eclampsia, stroke, cortical blindness or retinal detachment, hypertensive encephalopathy, systolic blood pressure ≥ 160 mmHg, myocardial infarction, intubation (other than for Cesarean section), pulmonary edema, platelets < 50 × 10^9^/L (without transfusion), disseminated intravascular coagulation, thrombotic thrombocytopenic purpura/hemolytic uremic syndrome, hepatic dysfunction (alanine transaminase ≥ 70 IU/L), hepatic hematoma or rupture, acute fatty liver of pregnancy, creatinine > 150 μmol/L, renal dialysis, placental abruption, major postpartum hemorrhage or major infection.

### Biomarker measurement

An initial panel of biomarkers was selected based on *a-priori* knowledge of an association with PE, a biological role in placentation or a role in cellular mechanisms involved in the pathogenesis of PE, e.g. angiogenesis, inflammation, coagulation. The full list of 47 biomarkers, measured with 57 assays (in which potentially biologically important assays of different epitope specificity were available) was generated following a review of the literature, appraisal of selected bibliography and consultation with medical experts (Table S1).

Samples were labeled and transported to the laboratory, where they were spun at 3000 rpm for 10 min. Plasma samples were tested for PlGF using the Triage PlGF Test (Alere Inc., San Diego, CA, USA) by trained laboratory staff at the study site where the sample was taken (as previously published). The additional 56 biomarker assays were analyzed in a central laboratory facility (Alere, San Diego, CA, USA) and full details of assay methods are given in Appendix S1 and Table S2. All participants were delivered and pregnancy outcomes recorded before biomarker concentrations were analyzed and revealed, and all laboratory staff were blinded to clinical outcomes.

### Statistical analysis

Standard distributional checks showed high levels of skewness for all 57 assays, which were consistent with underlying values of log normal distribution. Logged values of these biomarkers were therefore used. Before considering the pregnancy outcomes, statistical factor analysis of biomarker data was undertaken, reducing the 47 biomarkers into a smaller number of highly correlated groups, solely on the basis of the correlations between the biomarkers. Factor summary scores were then calculated for all the women. Consideration of scree plots and eigenvalues (> 2) identified the most important factors for further analysis^12^. These factors were rotated (orthogonal varimax method) so that each factor related strongly (correlation > 0.6) to a small number of biomarkers only (factor analysis displayed in Table S3).

The factor scores were entered into a multiple logistic regression model for prediction of subsequent SGA. Two factors (and their biomarkers), with significant odds ratios for prediction of SGA < 3^rd^ centile, were identified for further investigation (Tables S4 and S5). Stepwise logistic regression was used to determine which biomarkers appeared to provide additional information beyond that derived from PlGF, and prediction scores were extracted for the best combinations. A comparison of areas under the receiver–operating characteristics curves (AUCs) of individual biomarkers and combinations was made to see if any of the additional information was both consistent and clinically useful. Significance was assessed through the use of a non‐parametric test, which allowed for non‐independence of observations on the same participant, with Bonferroni correction for multiple testing.

Some biomarkers, with high uniqueness scores, were not strongly associated with any factor. To investigate whether any of these biomarkers had prognostic power in addition to that provided by PlGF and biomarkers identified earlier, stepwise logistic regression analysis was undertaken.

The best performing biomarker was then assessed using standard test performance indices (sensitivity, specificity, predictive values and AUC) against currently utilized ultrasound parameters in the subgroup of women with an ultrasound scan within 14 days of blood sampling, for the prediction of SGA and adverse perinatal outcome. A sensitivity analysis was conducted excluding those fetuses in which the scan on the day of enrollment had abnormal findings (AC or EFW < 10^th^ centile, oligohydramnios or absent/reversed end diastolic flow (*n* = 20)).

Statistical analysis was carried out in the statistical package Stata version 11.2 (College Station, TX, USA); statistical significance was taken as *P* < 0.05. The prespecified sample size was calculated for accurate estimation of the sensitivity (within 10%) and specificity (within 6%) of a biomarker, assuming a sensitivity of 0.90, specificity of 0.90 and two‐tailed 95% CIs, for determining the primary endpoint; this required 62 patients with PE and 150 women not meeting the primary endpoint.

The study is reported in accordance with Strengthening the Reporting of Observational Studies in Epidemiology guidelines (Table S6)^13^, it was approved by East London Research Ethics Committee (ref. 10/H0701/117) and it followed institutional guidelines.

## RESULTS

**Figure 1:**
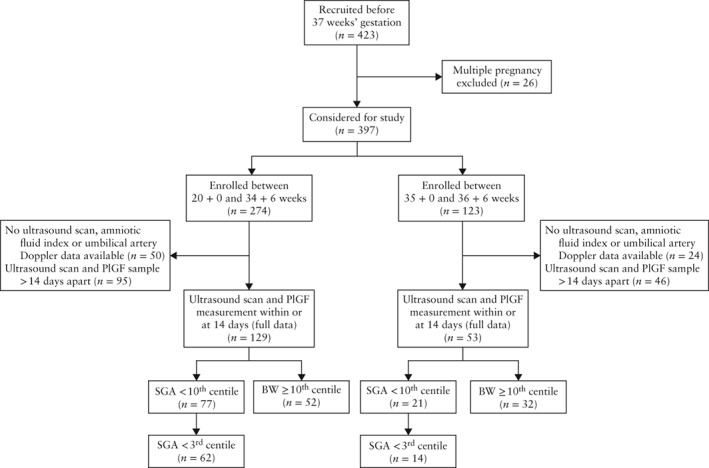
Flowchart of study participants. BW, birth weight; PlGF, placental growth factor; SGA, small‐for‐gestational age.

Between January 2011 and February 2012, 274 women presenting with suspected PE and a singleton pregnancy were enrolled between 20 + 0 and 34 + 6 weeks' gestation (Group 1), and 123 women were enrolled between 35 + 0 and 36 + 6 weeks (Group 2) (Figure 1).

The characteristics of the women in Group 1 at booking and enrollment are shown in Table 1 and details of maternal and neonatal outcomes are given in Table 2. Of the 274 women in Group 1, 96 (35%) delivered a SGA infant < 3^rd^ centile (SGA‐3) (of whom 90% developed PE) and 130 (47%) delivered a SGA infant < 10^th^ centile (of whom 81% developed PE). Adverse perinatal outcome was three times higher (39% *vs* 13%) in cases complicated by SGA‐3 than in those delivering an infant with appropriate‐for‐gestational‐age birth weight. Stillbirth occurred in six pregnancies, in five of which the birth weight was < 3^rd^ centile. In all stillbirth cases the PlGF concentration was < 5^th^ centile at enrollment, and predated the detection of ultrasound abnormalities by 7 to 39 days and the occurrence of stillbirth by 10 to 53 days.

**Table 1:** Characteristics of 274 women presenting at 20 + 0 to 34 + 6 weeks' gestation with suspected pre‐eclampsia (PE), according to subsequent birth weight of infant

Characteristic	Women with SGA infant < 3^rd^ centile (*n* = 96)	Women with SGA infant < 10^th^ centile (*n* = 130)	Women with infant ≥ 10^th^ centile (*n* = 144)
At booking			
Age (years)	31.9 (27.2–36.2)	31.9 (27.4–36.4)	31.7 (26.3–35.6)
BMI (kg/m^2^)	26.8 (24.1–31.2)	28.0 (23.9–32.8)	29.3 (24.7–34.9)
White ethnicity	63 (66)	87 (67)	92 (64)
Highest systolic BP (mmHg)	120 (110–130)	121 (110–130)	120 (110–130)
Highest diastolic BP (mmHg)	74 (65–81)	74 (65–81)	75 (68–82)
Smoker at booking	17 (18)	24 (18)	29 (20)
Quit smoking during pregnancy	10 (10)	14 (11)	19 (13)
Previous PE requiring delivery < 34 weeks	15 (16)	18 (14)	12 (8)
Chronic hypertension	11 (11)	21 (16)	23 (16)
At enrollment			
GA at sampling (weeks)	31.0 (27.6–33.0)	31.0 (27.6–33.1)	31.1 (28.0–33.6)
New‐onset hypertension	60 (63)	80 (62)	65 (45)
Worsening of underlying hypertension	16 (17)	24 (18)	32 (22)
New‐onset dipstick proteinuria	58 (60)	79 (61)	71 (49)
Highest systolic BP (mmHg)	147 (137–160)	148 (138–160)	141 (128–156)
Highest diastolic BP (mmHg)	94 (83–100)	94 (83–100)	90 (80–100)

Values given as median (interquartile range) or *n* (%).

BMI, body mass index; BP, blood pressure; GA, gestational age; SGA, small‐for‐gestational age.

**Table 2:** Delivery characteristics and maternal and neonatal outcome of 274 women presenting at 20 + 0 to 34 + 6 weeks' gestation with suspected pre‐eclampsia (PE), according to subsequent birth weight of infant

Characteristic	Women with SGA infant < 3^rd^ centile (*n* = 96)	Women with SGA infant < 10^th^ centile (*n* = 130)	Women with infant ≥ 10^th^ centile (*n* = 144)
Onset of labor*			
Spontaneous	3 (3)	7 (5)	32 (22)
Induced	29 (30)	42 (32)	64 (44)
Prelabor Cesarean section	64 (67)	80 (62)	46 (32)
Mode of delivery*			
Spontaneous vaginal	15 (16)	25 (19)	45 (31)
Assisted vaginal	5 (5)	8 (6)	21 (15)
Cesarean section	75 (78)	95 (73)	78 (54)
Adverse maternal outcome†	44 (46)	61 (47)	56 (39)
GA at delivery (weeks)	33.8 (30.8–36.1)	34.4 (31.4–37.3)	38.1 (36.0–39.4)
Fetal death	5 (5)	5 (4)	1 (1)
Neonatal death	2 (2)	2 (2)	0 (0)
Birth weight (g)	1537 (1043–1910)	1660 (1200–2310)	3128 (2698–3545)
SGA < 10^th^ birth‐weight centile	96 (100)	130 (100)	0 (0)
SGA < 3^rd^ birth‐weight centile	96 (100)	96 (74)	0 (0)
SGA < 1^st^ birth‐weight centile	68 (71)	68 (52)	0 (0)
Adverse perinatal outcome†	37 (39)	41 (32)	19 (13)
Maternal diagnosis			
No disease	0 (0)	1 (1)	21 (15)
Gestational hypertension	1 (1)	1 (1)	25 (17)
Chronic hypertension	4 (4)	12 (9)	16 (11)
PE	86 (90)	106 (82)	59 (41)
HELLP syndrome	1 (1)	1 (1)	1 (1)
Other	4 (4)	9 (7)	22 (15)

Values given as median (interquartile range) or *n* (%).

* Data missing for onset of labor in one woman and mode of delivery in two.

† Adverse outcome defined in main text.

GA, gestational age; SGA, small‐for‐gestational age.

The predictive performance of the most promising biomarkers as depicted by AUCs is shown in Table 3; AUCs for all 47 biomarkers measured are given in Table S7, and individual median biomarker concentrations in women sampled prior to 35 weeks are shown in Table S8. In isolation, PlGF had the best predictive performance for the detection of SGA‐3 when measured before 35 weeks' gestation, with an AUC of 0.83 (sensitivity, 89.7% (95% CI, 81.7–94.9%); specificity, 58.7% (95% CI, 51.1–66.0%); positive predictive value, 53.8% (95% CI, 45.7–61.7%); and negative predictive value (NPV), 91.3% (95% CI, 84.6–95.8%)). Combinations of the most promising biomarkers (Table 3) showed only minimal non‐significant increases in AUC for the prediction of SGA‐3 (from 0.83 to 0.84) and SGA‐10 (from 0.79 to 0.80).

**Table 3:** Performance of individual biomarkers and their combinations (derived from logistic regression analysis) for prediction of small‐for‐gestational‐age (SGA) < 3^rd^ centile and < 10^th^ centile in 274 women presenting at 20 + 0 to 34 + 6 weeks' gestation with suspected pre‐eclampsia (PE)

Biomarker	AUC (95% CI)		*P* *
	SGA < 3^rd^ centile	SGA < 10^th^ centile	
Nephrin	0.63 (0.56–0.70)	0.62 (0.55–0.69)	< 0.001
CPA‐4†	0.63 (0.57–0.70)	0.62 (0.55–0.68)	< 0.001
sFlt‐1	0.73 (0.67–0.79)	0.69 (0.63–0.76)	< 0.001
Endoglin	0.74 (0.68–0.80)	0.73 (0.67–0.79)	< 0.001
PlGF†	0.83 (0.78–0.88)	0.79 (0.73–0.84)	—
PlGF/s‐Flt ratio†	0.80 (0.75–0.85)	0.77 (0.71–0.82)	0.004
PlGF/endoglin ratio†	0.82 (0.77–0.86)	0.78 (0.73–0.83)	0.204
PlGF† + CPA‐4†	0.83 (0.78–0.88)	0.79 (0.74–0.84)	0.560
PlGF† + nephrin	0.84 (0.79–0.88)	0.80 (0.74–0.85)	0.475
PlGF† + nephrin + CPA‐4†	0.84 (0.79–0.89)	0.80 (0.74–0.85)	0.390

* Comparison of performance of single biomarker (or combination) *vs* placental growth factor (PlGF) alone.

† Low concentration of biomarker or low ratio correlates to disease.

AUC, area under receiver–operating characteristics curve; CPA‐4, carboxypeptidase A4 sFlt‐1, soluble fms‐like tyrosine kinase‐1.

Of women enrolled before 35 weeks, 129 had an ultrasound scan with all parameters recorded within 14 days of enrollment. The test performances of ultrasound parameters and PlGF (the best performing biomarker) for determining SGA‐3 and SGA‐10 are shown in Table 4 and Table S9, respectively, with PlGF alone having a higher sensitivity (SGA‐3, 93% (95% CI, 84–98%)) and NPV (SGA‐3, 90% (95% CI, 76–97%)) than any other indicator. While the addition of PlGF to currently used ultrasound parameters (AC or EFW < 10^th^ centile) increased the sensitivity for the detection of SGA‐3 (from 71% to 97%), the addition of ultrasound parameters to PlGF measurement did not markedly enhance sensitivity (from 93% to 97%). Adverse perinatal outcome (excluding SGA in this definition) occurred in 22% (60 of 274 infants). In predicting composite adverse perinatal outcome, PlGF had the highest sensitivity (90%) and NPV (90%) compared with all ultrasound measurements (*n* = 129; Table 5). In a sensitivity analysis, performance of the ultrasound and PlGF variables was similar when those with an abnormal scan on the day of enrollment were excluded from the analysis (Tables S10 and S11).

**Table 4:** Performance of individual indicators and their combinations for prediction of small‐for‐gestational‐age (SGA) < 3^rd^ customized birth‐weight centile in 129 women presenting at 20 + 0 to 34 + 6 weeks' gestation with suspected pre‐eclampsia, who underwent ultrasound examination within 14 days of enrollment

Indicator	Sensitivity (95% CI) (%)	Specificity (95% CI) (%)	PPV (95% CI) (%)	NPV (95% CI) (%)	LR+ (95% CI)	LR− (95% CI)
Indicator in isolation						
AC or EFW < 10^th^ centile	71.2 (57.9–82.2)	92.5 (83.4–97.5)	89.4 (76.9–96.5)	78.5 (67.8–86.9)	9.5 (4.0–22.5)	0.31 (0.21–0.47)
Oligohydramnios*	18.6 (9.7–30.9)	98.5 (92.0–100.0)	91.7 (61.5–99.8)	57.9 (48.3–67.1)	12.5 (1.7–93.9)	0.83 (0.73–0.94)
AREDF	20.3 (11.0–32.8)	98.5 (92.0–100.0)	92.3 (64.0–99.8)	58.4 (48.8–67.6)	13.6 (1.8–101.7)	0.81 (0.71–0.92)
PlGF < 100 pg/mL	93.2 (83.5–98.1)	52.2 (39.7–64.6)	63.2 (52.2–73.3)	89.7 (75.8–97.1)	2.0 (1.5–2.5)	0.13 (0.05–0.34)
Combinations of indicators						
AC or EFW < 10^th^ centileor oligohydramnios or AREDF	72.9 (59.7–83.6)	91.0 (81.5–96.6)	87.8 (75.2–95.4)	79.2 (68.5–87.6)	8.1 (3.7–17.7)	0.30 (0.19–0.46)
AC or EFW < 10^th^ centile or PlGF < 100 pg/mL	96.6 (88.3–99.6)	49.3 (36.8–61.8)	62.6 (51.9–72.6)	94.3 (80.8–99.3)	1.9 (1.5–2.3)	0.07 (0.02–0.28)

* Oligohydramnios defined as amniotic fluid index < 5^th^ centile for gestational age.

AC, abdominal circumference; AREDF, absent or reversed end‐diastolic flow in umbilical artery; EFW, estimated fetal weight; LR+/LR−, positive/negative likelihood ratio; NPV, negative predictive value; PlGF, placental growth factor; PPV, positive predictive value.

**Table 5:** Performance of individual indicators and their combinations for prediction of adverse perinatal outcome in 129 women presenting at 20 + 0 to 34 + 6 weeks' gestation with suspected pre‐eclampsia, who underwent ultrasound examination within 14 days of enrollment

Indicator	Sensitivity (95% CI) (%)	Specificity (95% CI) (%)	PPV (95% CI) (%)	NPV (95% CI) (%)	LR+ (95% CI)	LR− (95% CI)
Indicator in isolation						
AC or EFW < 10^th^ centile	48.7 (32.4–65.2)	67.8 (56.9–77.4)	40.4 (26.4–55.7)	74.7 (63.6–83.8)	1.5 (1.0–2.4)	0.76 (0.54–1.06)
Oligohydramnios*	12.8 (4.3–27.4)	92.0 (84.1–96.7)	41.7 (15.2–72.3)	70.2 (60.9–78.4)	1.6 (0.5–4.7)	0.95 (0.83–1.09)
AREDF	12.8 (4.3–27.4)	90.8 (82.7–95.9)	38.5 (13.9–68.4)	69.9 (60.6–78.2)	1.4 (0.5–4.0)	0.96 (0.84–1.10)
PlGF < 100 pg/mL	89.7 (75.8–97.1)	40.2 (29.9–51.3)	40.2 (29.9–51.3)	89.7 (75.8–97.1)	1.5 (1.2–1.8)	0.25 (0.10–0.67)
Combinations of indicators						
AC or EFW < 10^th^ centile or oligohydramnios or AREDF	53.8 (37.2–69.9)	67.8 (56.9–77.4)	42.9 (28.8–57.8)	76.6 (65.6–85.5)	1.7 (1.1–2.6)	0.68 (0.47–0.98)
AC or EFW < 10^th^ centile or PlGF < 100 pg/mL	92.3 (79.1–98.4)	36.8 (26.7–47.8)	39.6 (29.5–50.4)	91.4 (76.9–98.2)	1.5 (1.2–1.8)	0.21 (0.07–0.64)

* Oligohydramnios defined as amniotic fluid index < 5^th^ centile for gestational age.

AC, abdominal circumference, AREDF, absent or reversed end‐diastolic flow in umbilical artery; EFW, estimated fetal weight; LR+/LR−, positive/negative likelihood ratio; NPV, negative predictive value; PlGF, placental growth factor; PPV, positive predictive value.

A total of 123 women were enrolled between 35 + 0 and 36 + 6 weeks' gestation (Group 2); characteristics of these women at booking and enrollment and details of maternal and neonatal outcomes are described in Tables S12 and S13. AUCs for all 47 biomarkers measured between 35 + 0 and 36 + 6 weeks are given in Table S14. When measured in isolation, PlGF had an AUC of 0.69 for predicting SGA‐3 and 0.74 for SGA‐10; addition of carboxypeptidase A4 raised this to 0.77 for SGA‐3 and 0.81 for SGA‐10 (Table S15). The addition of other biomarkers yielded little benefit. In this group, PlGF had a higher sensitivity than all other currently used ultrasound indicators in predicting SGA infants (Tables S16 and S17) and adverse perinatal outcomes (Table S18).

## DISCUSSION

Our study shows that PlGF measurement has a high sensitivity and NPV in the determination of subsequent delivery of a SGA infant, and in the prediction of adverse perinatal outcome, in women presenting with suspected preterm PE. We evaluated SGA < 3^rd^ birth‐weight centile to identify fetuses more likely to be growth restricted, rather than constitutionally small. Our study suggests that PlGF measurement has a potential role alongside ultrasound assessment in the surveillance of high‐risk women with suspected PE and that integration of PlGF with current ultrasound parameters may increase detection rates of SGA. Ultrasound has an essential role in the detection of falling growth velocity, oligohydramnios and abnormal umbilical artery Doppler waveforms, which will continue to be used to stratify surveillance and time delivery appropriately. The use of PlGF for the prediction of SGA relates to this high‐risk group of women with suspected PE and cannot be generalized to low‐risk healthy pregnant women^14^.

Of 46 additional biomarkers evaluated in isolation or in combination with PlGF, there was minimal added value to the predictive performance of PlGF alone, and these markers are unlikely to be of utility in the clinical setting. It is possible that serial PlGF concentrations, with measurements made closer to outcome, may further improve the predictive ability while other biomarkers may only become significant closer to outcome. Placental pathology would have been a useful additional tool for assessing fetal growth restriction, but it was not available for this study.

A possible source of intervention bias is that ultrasound results were revealed to clinicians while biomarker results were not. At the time of the study in the UK, it was not common practice to deliver for falling growth velocity alone (i.e. pre‐empting delivery of a SGA infant) unless the EFW fell below the < 10^th^ centile. Adverse perinatal outcome (excluding SGA) was chosen as a secondary outcome to evaluate the performance of the variables on this additional clinically meaningful endpoint.

This study enrolled women who presented for obstetric assessment with a broad range of symptoms and signs of suspected PE, including those with underlying maternal disease. This is more informative than evaluating the tests against normal healthy pregnant women (as in a case–control study), as it is likely to reflect more closely the test performance in the usual clinical setting. The multicenter nature of the study, incorporating women of geographic and ethnic diversity, adds to the generalizability of the results. Further strengths of the study are that all final clinical diagnoses were adjudicated by a panel of medical experts and all clinical and laboratory staff were unaware of biomarker results until completion of the study.

It is a feature of our study that the assessments (including ultrasound examination) were performed in a local healthcare setting without referral, ultrasound or management protocols being dictated centrally by the research team. It is a strength that this pragmatic approach makes it likely that the prognostic variables would have comparable performance when translated beyond the research setting, with the findings directly generalizable to similar healthcare settings. However, it is a potential limitation that such an approach does not reflect assessment of ultrasound as undertaken in some healthcare systems (e.g. by a maternal–fetal medicine subspecialist).

The findings of this study relate to similar healthcare settings in which same‐day ultrasound assessment is not routinely undertaken for women presenting with suspected PE, owing to national guideline recommendations or lack of trained ultrasonographers. In settings in which all women with suspected PE undergo same‐day ultrasound assessment by a maternal–fetal medicine subspecialist, the performance of ultrasound may be different. As we included scans performed within 14 days after blood sampling, ultrasound scans may have been undertaken closer to the clinical endpoint (and would therefore not have been expected to introduce bias against ultrasound test performance).

We are not aware of any study that has compared such a wide panel of biomarkers (*n* = 47) for the prediction of subsequent SGA in women with suspected PE. Reports on the capability of PlGF to predict SGA have been conflicting. Initial small case–control studies in the first and second trimesters for the prediction of SGA found no significant relationship^15^, ^16^, but subsequent larger case–control studies and several prospective cohorts measuring PlGF in the second and first trimesters have reported an association between low PlGF concentrations and early‐onset pre‐eclampsia^17^, ^18^, stillbirth^19^ and SGA^20^, ^21^, ^22^. The few small (*n* = 21 or fewer), mainly case–control studies in which measurement was undertaken in the third trimester (including at time of delivery), generally concur with our finding of low PlGF concentration in women with subsequent delivery of a SGA infant^23^, ^24^, ^25^, ^26^, particularly those with significant underlying placental pathology^27^. As impaired placental function underpins a substantial proportion of cases of SGA (and PE)^28^, an angiogenic placental factor such as PlGF has biological plausibility for prediction. A recent systematic review of 53 studies (principally of first‐ and second‐trimester prediction, and with no studies of PlGF in a similar cohort to this study) investigated the value of biomarkers in the prediction of fetal growth restriction in singleton pregnancies and concluded that PlGF emerged as the most promising of the 37 biomarkers reported^6^. The finding that PlGF measurements also predict adverse perinatal outcome is supported by two other studies^29^, ^30^, but the first evaluated PlGF measurements in the first trimester while the second reported a combined maternal and perinatal adverse outcome.

SGA has the highest population‐attributable risk value (23%) for stillbirth of all pregnancy‐specific disorders^31^. In this study cohort, five of six cases complicated by stillbirth delivered an infant with a birth weight < 3^rd^ centile. In a setting in which ultrasound is not routinely performed in all women with suspected PE, PlGF measurement might facilitate earlier and more accurate detection of SGA associated with perinatal mortality, allowing appropriate surveillance of those at highest risk with the aim of improving outcome. Such a strategy could allow appropriate targeting of resources to at‐risk pregnancies with subsequent improvements in maternal and fetal outcomes.

## Supporting information


**Appendix S1** Biomarker assays.
**Table S1** List of biomarker abbreviations and units
**Table S2** Biomarker assay information
**Table S3** Results of factor analysis: loadings of biomarkers on five largest factors (eigenvalues > 2) after varimax rotation showing loadings > 0.6 only and uniqueness > 0.6
**Table S4** Odds ratios derived from multiple logistic regression analysis of the five factors for prediction of delivery of SGA infant in women presenting with suspected pre‐eclampsia before 35 weeks' gestation (odds ratios are for a change of 1 SD in the factor score). Factors 3 and 4 (with significant odds ratios for prediction of SGA infant < 3^rd^ centile) were taken forward for further analysis
**Table S5** Odds ratios derived from multiple logistic regression analysis of five factors for prediction of delivery of SGA infant in women presenting between 35 + 0 and 36 + 6 weeks' gestation with suspected pre‐eclampsia
**Table S6** STROBE checklist
**Table S7** ROC curve areas (with 95% CI) for individual biomarkers to predict small‐for‐gestational age (SGA) < 3^rd^ and < 10^th^ customized birth‐weight centiles in women presenting with suspected pre‐eclampsia before 35 weeks' gestation
**Table S8** Individual median biomarker concentrations (quartiles) in women presenting before 35 weeks' gestation with suspected pre‐eclampsia
**Table S9** Predictive performance of individual indicators and their combinations, to predict delivery of small‐for‐gestational age (SGA) < 10^th^ customized birth‐weight centile in 129 women presenting at 20 + 0 to 34 + 6 weeks' gestation with suspected pre‐eclampsia, who underwent ultrasound examination within 14 days of enrolment
**Table S10** Predictive performance of individual indicators and their combinations, to predict delivery of small‐for‐gestational age (SGA) < 3^rd^ customized birth‐weight centile in 109 women presenting at 20 + 0 to 34 + 6 weeks' gestation with suspected pre‐eclampsia, who underwent ultrasound examination within 14 days of enrolment, excluding those with known abnormal scan findings on day of enrolment
**Table S11** Test performance statistics for individual indicators and their combinations to predict adverse perinatal outcome in 109 women presenting before 35 weeks' gestation with suspected pre‐eclampsia, who underwent ultrasound examination within 14 days of enrolment, excluding those with known abnormal scan findings on day of enrolment
**Table S12** Characteristics of 123 women presenting between 35 + 0 and 36 + 6 weeks' gestation with suspected pre‐eclampsia (PE), according to subsequent birth weight of infant
**Table S13** Delivery characteristics and maternal and neonatal outcome of 123 women presenting between 35 + 0 and 36 + 6 weeks' gestation with suspected pre‐eclampsia (PE), according to subsequent birth weight of infant
**Table S14** Individual biomarker areas under receiver–operating characteristics curves (AUCs) when sampled between 35 + 0 and 36 + 6 weeks' gestation
**Table S15** Predictive performance of individual biomarkers and combinations (derived from logistic regression) for prediction of small‐for‐gestational age (SGA) < 3^rd^ centile and < 10^th^ centile in 123 women presenting between 35 + 0 and 36 + 6 weeks' gestation with suspected pre‐eclampsia
**Table S16** Predictive performance of individual indicators and their combinations, to predict small‐for‐gestational age (SGA) < 3^rd^ customized birth‐weight centile in 53 women presenting between 35 + 0 and 36 + 6 weeks' gestation with suspected pre‐eclampsia, who underwent ultrasound examination within 14 days of enrolment
**Table S17** Predictive performance of individual indicators and their combinations, to predict small‐for‐gestational age (SGA) < 10^th^ customized birth‐weight centile in 53 women presenting between 35 + 0 and 36 + 6 weeks' gestation with suspected pre‐eclampsia, who underwent ultrasound examination within 14 days of enrolment
**Table S18** Predictive performance of individual indicators and their combinations, to predict adverse perinatal outcome in 53 women presenting between 35 + 0 and 36 + 6 weeks' gestation with suspected pre‐eclampsia, who underwent ultrasound examination within 14 days of enrolment
