# Supplementary material for: Predicting delivery of a small‐for‐gestational‐age infant and adverse perinatal outcome in women with suspected pre‐eclampsia
Source: Ultrasound Obstet Gynecol. 2018 Feb 7;51(3):387–95. doi: 10.1002/uog.17490 (PMC5887913; doi:10.1002/uog.17490)
Supplement: Supplementary file 1 — Appendix S1 Biomarker assays. Table S1 List of biomarker abbreviations and units Table S2 Biomarker assay information Table S3 Results of factor analysis: loadings of biomarkers on five largest factors (eigenvalues > 2) after varimax rotation showing loadings > 0.6 only and uniqueness > 0.6 Table S4 Odds ratios derived from multiple logistic regression analysis of the five factors for prediction of delivery of SGA infant in women presenting with suspected pre‐eclampsia before 35 weeks' gestation (odds ratios are for a change of 1 SD in the factor score). Factors 3 and 4 (with significant odds ratios for prediction of SGA infant < 3rd centile) were taken forward for further analysis Table S5 Odds ratios derived from multiple logistic regression analysis of five factors for prediction of delivery of SGA infant in women presenting between 35 + 0 and 36 + 6 weeks' gestation with suspected pre‐eclampsia Table S6 STROBE checklist Table S7 ROC curve areas (with 95% CI) for individual biomarkers to predict small‐for‐gestational age (SGA) < 3rd and < 10th customized birth‐weight centiles in women presenting with suspected pre‐eclampsia before 35 weeks' gestation Table S8 Individual median biomarker concentrations (quartiles) in women presenting before 35 weeks' gestation with suspected pre‐eclampsia Table S9 Predictive performance of individual indicators and their combinations, to predict delivery of small‐for‐gestational age (SGA) < 10th customized birth‐weight centile in 129 women presenting at 20 + 0 to 34 + 6 weeks' gestation with suspected pre‐eclampsia, who underwent ultrasound examination within 14 days of enrolment Table S10 Predictive performance of individual indicators and their combinations, to predict delivery of small‐for‐gestational age (SGA) < 3rd customized birth‐weight centile in 109 women presenting at 20 + 0 to 34 + 6 weeks' gestation with suspected pre‐eclampsia, who underwent ultrasound examination within 14 days of enrolment, excluding those with know [file UOG-51-387-s001.docx]

**Supplemental digital content to**

**Predicting delivery of a small-for-gestational age infant in women with suspected preeclampsia**

**Table S1: List of Biomarker abbreviations and units**

| Biomarker | Biomarker full name | Units |
| --- | --- | --- |
| ADAM 9 | Disintegrin and metalloproteinase domain-containing protein 9 | pg/ml |
| Angiogenin | Angiogenin | μg/ml |
| ANP | Natriuretic peptide A | ng/ml |
| Arginase-1 | Arginase 1 | ng/ml |
| Arginase-2 | Arginase 2 | ng/ml |
| BNP | Brain natriuretic peptide | ng/ml |
| Caspase | Caspase | ng/ml |
| CCL23 | MIP3, C motif chemokine 23 | ng/ml |
| C-Met-109a | Tyrosine kinase | ng/ml |
| C-Met-111a | Tyrosine kinase | ng/ml |
| CPA-4 | Carboxypeptidase A4 | ng/ml |
| CRP | C reactive protein | μg/ml |
| CXCL10 | CXC motif chemokine 10 | ng/ml |
| Cystatin | Cystatin | ng/ml |
| Elafin-131 | Elafin | ng/ml |
| Elafin-132 | Elafin | ng/ml |
| Endoglin | Endoglin | ng/ml |
| Endothelin | Endothelin | pg/ml |
| Ephrin | Ephrin | pg/ml |
| ESAM-1 | Endothelial Cell-selective adhesion molecule | ng/ml |
| FAS | Tumour necrosis factor receptor superfamily member 6 | ng/ml |
| FasL | Tumour necrosis factor ligand superfamily member 6 | ng/ml |
| FIH | Hypoxia inducible factor 1-alpha inhibitor | ng/ml |
| HbF | Haemoglobin Fetal | ng/ml |
| ICAM-1 | Intercellular adhesion molecule 1 | ng/ml |
| IL-1ra | Interleukin 1 receptor antagonist | pg/ml |
| Kunitz-2 (HAI-2) 34a | Kunitz-type protease inhibitor 2 | ng/ml |
| Kunitz-2 (HAI-2) 35b | Kunitz-type protease inhibitor 2 | ng/ml |
| Kunitz-2 (HAI-2) 40b | Kunitz-type protease inhibitor 2 | ng/ml |
| Leptin | Leptin | ng/ml |
| Leptin receptor | Leptin receptor | ng/ml |
| MIF-49a | Macrophage migration inhibitory factor | ng/ml |
| MIF-49b | Macrophage migration inhibitory factor | ng/ml |
| MMP-9 | Matrix metalloproteinase-9 | ng/ml |
| Nephrin-100a | Nephrin | ng/ml |
| Nephrin-101a | Nephrin | ng/ml |
| NGAL | Neutrophil gelatinase-associated lipocalin | ng/ml |
| PAI-1 | Plasminogen activator inhibitor 1 | ng/ml |
| PAI-2 | Plasminogen activator inhibitor 2 | ng/ml |
| PAPP-A | Pregnancy specific plasma protein A | ng/ml |
| PCT-95a | Procalcitonin | pg/ml |
| PCT-99b | Procalcitonin | pg/ml |
| Pentraxin-3-64a | Pentraxin-related protein PTX3 | ng/ml |
| Pentraxin-3-67a | Pentraxin-related protein PTX3 | ng/ml |
| Periostin | Periostin | ng/ml |
| PlGF | Placental Growth Factor | pg/ml |
| Podocalyxin | Podocalyxin | ng/ml |
| sFlt-1 | Soluble fms-like tyrosine kinase-1 | ng/ml |
| ST2-116b | Interleukin-1 receptor-like 1 | ng/ml |
| ST2-75b | Interleukin-1 receptor-like 1 | ng/ml |
| TGFβ-1 | Transforming growth factor beta-1 | ng/ml |
| TIMP-1 | Metelloproteinase Inhibitor 1 | ng/ml |
| TNFR1A | Tumour necrosis factor receptor superfamily member 1A | ng/ml |
| VEGF-C | Vascular endothelial growth factor C | ng/ml |
| Visfatin | Visfatin | ng/ml |
| WAP4C-HE4-85b | WAP four disulfide core domain protein 2 | ng/ml |
| WAP4C-HE4-91a | WAP four disulfide core domain protein 2 | ng/ml |

**Appendix S1**

The additional 57 biomarker assays were analysed in a central laboratory facility (Alere, San Diego, CA) at room temperature, following development of the assays to determine optimal formatting (i.e. sandwich format, competitive, diluted format, single analyte) with customisation of the Luminex assays to perform with each other on a single format, usually as a non-diluted neat assay optimized for each analyte. A list of biomarker assay information (low and high cut-offs, assay coefficient variable and assay format) is given in table S2.

The Luminex sandwich assays used a mouse-derived recombinant Fab conjugated to a magnetic bead as the capture and a biotin-conjugated recombinant Fab as the assay detection, optimised for use in Luminex xMap technology (Alere, San Diego). Recombinant Fab conjugated magnetic beads were added to the plate and washed. Sample was then incubated with the beads, followed by incubation with the detection antibody. The plate was washed, incubated with streptavidin-labeled phycoerythrin, washed and then read using a Luminex 200 reader. The Luminex competitive assay format used a recombinant Fab conjugated to the bead and a biotin-conjugated antigen as the assay detection. Recombinant Fab conjugated magnetic beads were added to the plate and washed. The sample and detection reagent were premixed, added simultaneously and then incubated. The final steps were as described for the Luminex sandwich assay.

The micro-titer ELISA assays used a streptavidin coated plate and biotin or fluorescein conjugated recombinant Fabs. The ELISA sandwich assay used a biotin-conjugated recombinant Fab as the capture and a fluorescein-conjugated recombinant Fab as the detection antibody. Capture antibody was coated on the plate, incubated, washed and sample added. After sample incubation, the plate was washed and then incubated with detection antibody. Following washing, the plate was incubated with anti-fluorescein antibody conjugated to alkaline phosphatase, washed, fluorescent substrate added and then read using a Tecan infinite F200 reader.

The ELISA competitive assay used a biotin-conjugated antigen as the capture and a fluorescein-conjugated recombinant Fab as the detection antibody. The plate was coated with capture antigen, incubated and washed. Addition of sample was immediately followed by addition of the detection antibody and incubated. The final steps were the same as the ELISA sandwich.

Each assay used an eight-point dose curve prepared gravimetrically in EDTA plasma or buffer. Immunoassays utilizing human plasma were performed in 384-well microtitre plates using Perkin-Elmer Minitrak robotic liquid handling system for all liquid handling steps. Test samples were added to the 384-well plate, containing wells for a calibration curve consisting of multiple analyte concentrations and control samples. Calibration curves were prepared gravimetrically in plasma from healthy donors. For sandwich assays, one concentration in each set of calibrators included neutralizing antibody for correction of endogenous antigen present in the plasma pool.

**Table S2: Biomarker assay information**

| Biomarker | Low Cutoff | High Cutoff | Assay Coefficient Variable | Assay format |
| --- | --- | --- | --- | --- |
| PlGF | 12 | 3000 | 13 | Sandwich, Luminex |
| ADAM-9 | 38.07 | 7913.74 | 11 | Sandwich, Luminex |
| Angiogenin | 0.14 | 61.24 | 7 | Competitive, Luminex |
| ANP | 0.048 | 71.93 | 13 | Sandwich, Luminex |
| Arginase-1 | 0.035 | 30.50 | 9 | Sandwich, Luminex |
| Arginase-2 | 1.318 | 378.14 | 15 | Sandwich, Luminex |
| BNP | 0.007 | 5.83 | 18 | Sandwich, Luminex |
| Caspase | 0.292 | 114.29 | 13 | Sandwich, Luminex |
| CCL23 | 0.009 | 3.52 | 7 | Sandwich, Luminex |
| C-Met-109a | 7.999 | 453.54 | 11 | Sandwich, Luminex |
| C-Met-111a | 77.147 | 1035.48 | 7 | Sandwich, Luminex |
| CPA-4 | 0.119 | 19.14 | 5 | Sandwich, Luminex |
| CRP | 0.07871 | 141.96 | 5 | Competitive, Luminex |
| CXCL10 | 0.006 | 5.81 | 10 | Sandwich, Luminex |
| Cystatin | 165.009 | 9072.87 | 21 | Competitive, Microtitre |
| Elafin-131 | 28.670 | 42668.61 | 10 | Competitive, Luminex |
| Elafin-132 | 21.159 | 42668.61 | 5 | Competitive, Luminex |
| Endoglin | 1.981 | 654.84 | 18 | Sandwich, Microtitre |
| Endothelin-1 | 0.704 | 901.9 | 13 | Sandwich, Luminex |
| Ephrin | 43.71 | 4009.97 | 20 | Sandwich, Luminex |
| ESAM-1 | 1.073 | 32.77 | 9 | Sandwich, Luminex |
| FAS | 0.115 | 152.86 | 22 | Sandwich, Luminex |
| FasL | 0.156 | 30.20 | 11 | Sandwich, Luminex |
| FIH | 0.003 | 18.50 | 6 | Sandwich, Luminex |
| HbF | 0.848 | 386.32 | 18 | Sandwich, Microtitre |
| ICAM-1 | 106.275 | 30231.73 | 6 | Competitive, Luminex |
| IL-1ra | 0.477 | 1434.20 | 9 | Sandwich, Luminex |
| Kunitz-2 (HAI-2) 34a | 0.016 | 10.17 | 19 | Sandwich, Luminex |
| Kunitz-2 (HAI-2) 35b | 0.140 | 57.15 | 13 | Sandwich, Luminex |
| Kunitz-2 (HAI-2) 40b | 0.159 | 57.38 | 7 | Sandwich, Luminex |
| Leptin | 5.509 | 148.56 | 11 | Sandwich, Luminex |
| Leptin receptor | 2.244 | 1079.61 | 9 | Sandwich, Luminex |
| MIF-49a | 3.912 | 25.99 | 10 | Sandwich, Luminex |
| MIF-49b | 0.414 | 70.85 | 9 | Sandwich, Luminex |
| MMP-9 | 4.542 | 202.82 | 5 | Sandwich, Luminex |
| Nephrin-100a | 0.517 | 19.98 | 25 | Sandwich, Luminex |
| Nephrin-101a | 0.094 | 19.74 | 22 | Sandwich, Luminex |
| NGAL | 0.625 | 2924.00 | 22 | Sandwich, Microtitre |
| PAI-1 | 0.194 | 103.48 | 12 | Sandwich, Luminex |
| PAI-2 | 0.047 | 77.90 | 5 | Sandwich, Luminex |
| PAPP-A | 0.189 | 812.10 | 7 | Sandwich, Luminex |
| PCT-95a | 12.22 | 9165.34 | 14 | Sandwich, Luminex |
| PCT-99b | 9.55 | 3982.50 | 14 | Sandwich, Luminex |
| Pentraxin-3-64a | 0.221 | 91.01 | 10 | Sandwich, Luminex |
| Pentraxin-3-67a | 0.940 | 59.41 | 16 | Sandwich, Luminex |
| Periostin | 0.538 | 107.38 | 7 | Sandwich, Luminex |
| Podocalyxin | 0.075 | 20.79 | 15 | Sandwich, Luminex |
| sFlt-1 | 0.006 | 27.86 | 10 | Sandwich, Luminex |
| ST2-116b | 0.038 | 21.25 | 14 | Sandwich, Luminex |
| ST2-75b | 0.075 | 44.63 | 6 | Sandwich, Luminex |
| TGFβ-1 | 0.040 | 63.98 | 9 | Sandwich, Luminex |
| TIMP-1 | 9.127 | 1917.45 | 5 | Competitive, Luminex |
| TNFR-1A | 0.230 | 31.02 | 18 | Sandwich, Luminex |
| VEGF-C | 0.527 | 74.07 | 5 | Sandwich, Luminex |
| Visfatin | 2.535 | 1738.71 | 13 | Sandwich, Luminex |
| WAP4C-HE4-85b | 0.129 | 89.71 | 11 | Sandwich, Luminex |
| WAP4C-HE4-91a | 1.516 | 54.27 | 6 | Sandwich, Luminex |

**Table S3: Results of factor analysis: loadings of biomarkers on five largest factors (eigenvalues > 2) after varimax rotation showing loadings > 0.6 only and uniqueness > 0.6**

| Biomarker | Factor 1 | Factor 2 | Factor 3 | Factor 4 | Factor 5 | Uniqueness |
| --- | --- | --- | --- | --- | --- | --- |
| ADAM-9 |  | 0.87 |  |  |  | 0.22 |
| Angiogenin |  |  |  |  | 0.62 | 0.41 |
| ANP | 0.69 |  |  |  |  | 0.36 |
| Arginase 1 | 0.63 |  |  |  |  | 0.5 |
| Arginase 2 |  |  | 0.81 |  |  | 0.18 |
| BNP |  |  | 0.67 |  |  | 0.37 |
| Caspase |  |  |  |  | 0.68 | 0.28 |
| CCL23 | 0.65 |  |  |  |  | 0.51 |
| CPA-4 | 0.62 |  |  |  |  | 0.51 |
| CRP |  |  |  |  |  | 0.85 |
| CXCL10 |  |  |  |  |  | 0.65 |
| Cystatin C |  |  |  |  |  | 0.87 |
| Elafin 131 |  |  |  |  |  | 0.7 |
| Elafin 132 |  |  |  |  |  | 0.91 |
| Endoglin |  |  |  | 0.74 |  | 0.41 |
| Ephrin |  | 0.78 |  |  |  | 0.34 |
| ESAM-1 | 0.76 |  |  |  |  | 0.35 |
| FAS | 0.61 |  |  |  |  | 0.48 |
| FasL |  | 0.8 |  |  |  | 0.28 |
| FIH |  |  |  |  | 0.73 | 0.42 |
| HbF |  |  |  |  |  | 0.96 |
| ICAM1 |  |  |  |  | 0.62 | 0.55 |
| Kunitz-2 (HAI-2) 34a | 0.67 |  |  |  |  | 0.41 |
| Kunitz-2 (HAI-2) 35b |  | 0.64 |  |  |  | 0.37 |
| Kunitz-2 (HAI-2) 40b |  | 0.74 |  |  |  | 0.2 |
| Leptin |  |  |  |  |  | 0.68 |
| MIF 49b |  |  |  |  | 0.63 | 0.45 |
| MMP-9 |  |  |  |  |  | 0.64 |
| Nephrin 100a |  | 0.73 |  |  |  | 0.35 |
| Nephrin 101a |  |  | 0.89 |  |  | 0.13 |
| NGAL |  |  |  |  |  | 0.71 |
| PAI-1 |  | 0.66 |  |  |  | 0.37 |
| PAPP-A |  |  |  |  |  | 0.73 |
| PCT 95a |  |  | 0.85 |  |  | 0.21 |
| PCT 99b |  |  |  |  |  | 0.61 |
| Pentraxin 3 64a |  | 0.73 |  |  |  | 0.31 |
| Pentraxin 3 67a |  |  | 0.7 |  |  | 0.31 |
| Periostin |  |  |  |  |  | 0.68 |
| PlGF |  |  |  | 0.83 |  | 0.31 |
| Podocalyxin |  |  | 0.81 |  |  | 0.19 |
| sFlt-1 |  |  |  | 0.82 |  | 0.16 |
| ST2 116 |  |  |  |  |  | 0.66 |
| TGFβ-1 | 0.72 |  |  |  |  | 0.37 |
| TIMP-1 |  |  |  |  | 0.7 | 0.41 |
| TNFR-1A | 0.75 |  |  |  |  | 0.31 |
| VEGF-C |  |  |  |  |  | 0.82 |
| Visfatin |  |  |  |  |  | 0.75 |
| WAP4C HE4 85b | 0.63 |  |  |  |  | 0.31 |
| WAP4C HE4 91a | 0.72 |  |  |  |  | 0.38 |

**Table S4: Odds ratios derived from multiple logistic regression analysis of the five factors in women presenting before 35 weeks’ gestation (odds ratios are for a change of 1 SD in the factor score). Factors 3 and 4 (with significant odds ratios for prediction of SGA infant <3^rd^ centile) were taken forward for further analysis**

| Factor | Biomarkers contained in factor | Women with SGA infant <3rd centile | Women with SGA infant <10th centile |
| --- | --- | --- | --- |
|  |  | Odds Ratio  (95% CI) | Odds Ratio  (95% CI) |
| 1 | ANP, Arginase-1, CCL23, CPA-4, ESAM-1, FAS, Kunitz-2, TGFBeta-1, TNFR-1A, WAP4C-HE4-85b, WAP4C-HE4-91a | 0.85 (0.64 - 1.13) | 0.89 (0.68 - 1.16) |
| 2 | ADAM-9, Ephrin, FasL, Kunitz 35b, Kunitz 40b, Nephrin, PAI-1, Pentraxin-3-64a | 0.79 (0.57 - 1.1) | 0.78 (0.58 - 1.03) |
| 3 | Arginase-2, BNP, Nephrin, PCT-95a, Pentraxin 3-67a, Podocalyxin | 1.67 (1.23 - 2.28) | 1.57 (1.18 - 2.07) |
| 4 | PlGF, Endoglin, sFlt-1 | 2.85 (2.13 - 3.82) | 2.38 (1.84 - 3.08) |
| 5 | Angiogenin, Caspase, FIH, ICAM-1, MIF, TIMP-1 | 1.04 (0.76 - 1.41) | 0.99 (0.74 - 1.31) |

**Table S5: Odds ratios (95%CI) derived from multiple logistic regression analysis of the five factors in women presenting between 35+0 and 36+6 weeks’ gestation (odds ratios are for a change of 1 SD in the factor score).**

| Factor | Biomarkers contained in factor | Women with SGA infant <3rd centile | Women with SGA infant <10th centile |
| --- | --- | --- | --- |
|  |  | Odds Ratio  (95% CI) | Odds Ratio  (95% CI) |
| 1 | ANP, Arginase-1, CCL23, CPA-4, ESAM-1, FAS, Kunitz-2, TGFBeta-1, TNFR-1A, WAP4C-HE4-85b, WAP4C-HE4-91a | 0.46 (0.26 - 0.78) | 0.42 (0.25 - 0.72) |
| 2 | ADAM-9, Ephrin, FasL, Kunitz 35b, Kunitz 40b, Nephrin, PAI-1, Pentraxin-3-64a | 0.97 (0.36 - 2.56) | 0.54 (0.21 - 1.41) |
| 3 | Arginase-2, BNP, Nephrin, PCT-95a, Pentraxin 3-67a, Podocalyxin | 1.80 (1.03 - 3.16) | 1.73 (1.02 - 2.94) |
| 4 | PlGF, Endoglin, sFlt-1 | 3.54 (1.52 - 8.26) | 6.89 (2.88 - 16.4) |
| 5 | Angiogenin, Caspase, FIH, ICAM-1, MIF, TIMP-1 | 1.07 (0.61 - 1.90) | 0.85 (0.50 - 1.45) |

**Text S6: STROBE checklist**

|  | Item | Information | Section/ Paragraph |
| --- | --- | --- | --- |
| Title and Abstract | 1 | Indicate the study’s design in the title or the Abstract | Abstract |
|  |  | Provide in the Abstract an informative and balanced summary of what was done and what was found | Abstract |
| Introduction |  |  |  |
| Background/ rationale | 2 | Explain the scientific background and rationale for the investigation being reported | Introduction |
| Objectives | 3 | State specific objectives, including any pre-specified hypotheses | Introduction |
| Methods |  |  |  |
| Study design | 4 | Present key elements of study design early in the paper | Participants |
| Setting | 5 | Describe the setting, locations, and relevant dates, including periods of recruitment, exposure, follow-up, and data collection | Participants |
| Participants | 6 | Give the eligibility criteria, and the sources and methods of selection of participants. | Participants |
| Variables | 7 | Clearly define all outcomes, exposures, predic-rs, potential confounders, and effect modifiers. | Methods |
| Data sources/ measurement | 8 | For each variable of interest, give sources of data and details of methods of assessment (measurement). | Biomarker measurement |
| Bias | 9 | Describe any efforts to address potential sources of bias | Biomarker measurement |
| Study size | 10 | Explain how the study size was arrived at | Statistical analysis |
| Quantitative variables | 11 | Explain how quantitative variables were handled in the analyses. | Statistical analysis |
| Statistical methods | 12 | Describe all statistical methods | Statistical analysis |
|  |  | Describe any methods used to examine subgroups and interactions | N/A |
|  |  | Explain how missing data were addressed | N/A |
|  |  | Describe any sensitivity analyses | N/A |
| **Results** |  |  |  |
| Participants | 13* | (a) Report numbers of individuals at each stage of study—eg numbers potentially eligible, examined for eligibility, confirmed eligible, included in the study, completing follow-up, and analysed | Figure 1 |
|  |  | (b) Give reasons for non-participation at each stage | Figure 1 |
|  |  | (c) Consider use of a flow diagram | Figure 1 |

| Descriptive data | 14* | (a) Give characteristics of study participants (eg demographic, clinical, social) and information on exposures and potential confounders | Table 1 and S11 |
| --- | --- | --- | --- |
|  |  | (b) Indicate number of participants with missing data for each variable of interest | N/A |
|  |  | (c) Summarise follow-up time (eg, average and total amount) | To delivery |
| Outcome data | 15* | Report numbers of outcome events or summary measures over time | Table 2 |
| Main results | 16 | (*a*) Give unadjusted estimates and, if applicable, confounder-adjusted estimates and their precision (eg, 95% confidence interval). Make clear which confounders were adjusted for and why they were included | N/A |
|  |  | (*b*) Report category boundaries when continuous variables were categorized | N/A |
|  |  | (*c*) If relevant, consider translating estimates of relative risk into absolute risk for a meaningful time period | N/A |
| Other analyses | 17 | Report other analyses done—eg analyses of subgroups and interactions, and sensitivity analyses | Tables 3-5 |
| **Discussion** |  |  |  |
| Key results | 18 | Summarise key results with reference to study objectives | Para 1 |
| Limitations | 19 | Discuss limitations of the study, taking into account sources of potential bias or imprecision. Discuss both direction and magnitude of any potential bias | Para 2 |
| Interpretation | 20 | Give a cautious overall interpretation of results considering objectives, limitations, multiplicity of analyses, results from similar studies, and other relevant evidence | Para 3-4 |
| Generalisability | 21 | Discuss the generalisability (external validity) of the study results | Para 3-4 |
| **Other information** |  |  |  |
| Funding | 22 | Give the source of funding and the role of the funders for the present study and, if applicable, for the original study on which the present article is based | Disclosures |

**Table S7: ROC curve areas (with 95% confidence intervals) for individual biomarkers to predict small-for-gestational age (SGA) <3rd and <10th customized birth-weight centiles in women presenting before 35 weeks’ gestation**

|  | Women with SGA infant <3rd centile | Women with SGA infant <10th centile |
| --- | --- | --- |
| ADAM-9 | 0.52 (0.48 - 0.56) | 0.54 (0.50 - 0.58) |
| Angiogenin | 0.59 (0.51 - 0.66) | 0.58 (0.51 - 0.64) |
| ANP | 0.56 (0.49 - 0.64) | 0.56 (0.49 - 0.63) |
| Arginase 1 | 0.51 (0.44 - 0.58) | 0.51 (0.44 - 0.58) |
| [Arginase 2] | 0.64 (0.56 - 0.71) | 0.63 (0.57 - 0.70) |
| BNP | 0.69 (0.62 - 0.75) | 0.64 (0.58 - 0.71) |
| [CCL23] | 0.57 (0.50 - 0.64) | 0.58 (0.51 - 0.65) |
| CRP | 0.58 (0.51 - 0.65) | 0.55 (0.48 - 0.62) |
| [CPA-4] | 0.63 (0.57 - 0.70) | 0.62 (0.55 - 0.68) |
| Caspase | 0.56 (0.49 - 0.63) | 0.56 (0.49 - 0.62) |
| CXCL10 | 0.51 (0.44 - 0.59) | 0.53 (0.46 - 0.59) |
| Cystatin C | 0.58 (0.51 - 0.65) | 0.59 (0.52 - 0.66) |
| [C-Met 109a] | 0.59 (0.52 - 0.66) | 0.58 (0.51 - 0.64) |
| [C-Met 111a] | 0.61 (0.54 - 0.68) | 0.59 (0.52 - 0.65) |
| Elafin 131 | 0.57 (0.50 - 0.64) | 0.54 (0.47 - 0.61) |
| Elafin 132 | 0.48 (0.41 - 0.55) | 0.47 (0.41 - 0.54) |
| Endoglin | 0.74 (0.68 - 0.80) | 0.73 (0.67 - 0.79) |
| [ESAM-1] | 0.55 (0.48 - 0.63) | 0.55 (0.48 - 0.62) |
| [Endothelin] | 0.53 (0.45 - 0.60) | 0.54 (0.48 - 0.61) |
| [Ephrin] | 0.49 (0.46 - 0.51) | 0.49 (0.47 - 0.51) |
| [FAS] | 0.53 (0.45 - 0.60) | 0.49 (0.42 - 0.56) |
| [FasL] | 0.52 (0.46 - 0.58) | 0.54 (0.48 - 0.59) |
| FIH | 0.55 (0.48 - 0.62) | 0.56 (0.49 - 0.62) |
| [HbF] | 0.44 (0.37 - 0.51) | 0.45 (0.38 - 0.52) |
| ICAM-1 | 0.57 (0.50 - 0.64) | 0.55 (0.48 - 0.62) |
| [IL-1ra] | 0.58 (0.51 - 0.65) | 0.59 (0.53 - 0.66) |
| Kunitz-2 (HAI-2) 34a | 0.52 (0.45 - 0.59) | 0.51 (0.44 - 0.58) |
| [Kunitz-2 (HAI-2) 35b] | 0.57 (0.50 - 0.64) | 0.56 (0.49 - 0.63) |
| [Kunitz-2 (HAI-2) 40b] | 0.58 (0.51 - 0.65) | 0.60 (0.53 - 0.66) |
| [Leptin] | 0.47 (0.40 - 0.54) | 0.49 (0.42 - 0.56) |
| [Leptin receptor] | 0.59 (0.52 - 0.66) | 0.56 (0.49 - 0.63) |
| [MIF -49a] | 0.54 (0.47 - 0.62) | 0.58 (0.51 - 0.65) |
| [MIF- 49b] | 0.55 (0.47 - 0.62) | 0.54 (0.47 - 0.61) |
| [MMP-9] | 0.52 (0.44 - 0.59) | 0.53 (0.46 - 0.60) |
| Nephrin 100a | 0.55 (0.50 - 0.60) | 0.55 (0.50 - 0.60) |
| Nephrin 101a | 0.63 (0.56 - 0.70) | 0.62 (0.55 - 0.69) |
| NGAL | 0.62 (0.55 - 0.69) | 0.60 (0.54 - 0.67) |
| [PAPP-A] | 0.60 (0.53 - 0.67) | 0.64 (0.57 - 0.70) |
| Pentraxin- 3 64a | 0.49 (0.44 - 0.55) | 0.48 (0.43 - 0.53) |
| Pentraxin- 3 67a | 0.64 (0.57 - 0.71) | 0.63 (0.56 - 0.69) |
| Periostin | 0.56 (0.48 - 0.63) | 0.54 (0.47 - 0.61) |
| PAI-1 | 0.53 (0.46 - 0.60) | 0.50 (0.43 - 0.57) |
| [PAI-2] | 0.68 (0.62 - 0.75) | 0.66 (0.59 - 0.72) |
| [PlGF] | 0.83 (0.78 - 0.88) | 0.79 (0.73 - 0.84) |
| Podocalyxin | 0.62 (0.55 - 0.69) | 0.59 (0.53 - 0.66) |
| PCT 95a | 0.67 (0.61 - 0.74) | 0.64 (0.57 - 0.71) |
| PCT 99b | 0.51 (0.44 - 0.58) | 0.52 (0.45 - 0.59) |
| sFlt-1 | 0.73 (0.66 - 0.79) | 0.69 (0.63 - 0.76) |
| ST2-116b | 0.62 (0.55 - 0.69) | 0.60 (0.53 - 0.66) |
| ST2 -75b | 0.59 (0.52 - 0.66) | 0.57 (0.51 - 0.64) |
| [TGFβ-1] | 0.60 (0.53 - 0.68) | 0.59 (0.52 - 0.66) |
| TIMP-1 | 0.60 (0.53 - 0.67) | 0.56 (0.49 - 0.63) |
| [TNFR-1A] | 0.54 (0.46 - 0.62) | 0.51 (0.44 - 0.58) |
| VEGF-C | 0.57 (0.50 - 0.65) | 0.58 (0.51 - 0.64) |
| Visfatin | 0.56 (0.49 - 0.63) | 0.56 (0.49 - 0.62) |
| WAP4C HE4 85b | 0.62 (0.55 - 0.69) | 0.60 (0.53 - 0.66) |
| WAP4C HE4 91a | 0.55 (0.48 - 0.63) | 0.57 (0.51 - 0.64) |

**Table S8: Individual median biomarker concentrations (quartiles) in women presenting before 35 weeks’ gestation**

| Biomarkers | Women with SGA infant <3^rd^ centile  (n= 96) | Women with SGA infant <10^th^ centile (n=130) | Women with infant ≥ 10^th^ centile  (n=144) |
| --- | --- | --- | --- |
| ADAM 9 * | 89.6% below limit of detection | 91.5% below limit of detection | 84.0% below limit of detection |
| Angiogenin (μg/ml) | 11.2  (7.71 - 19.3) | 11.2  (7.56 - 18.7) | 9.44  (6.56 - 15.1) |
| ANP (ng/ml) | 1.28  (0.52 - 3.39) | 1.06  (0.53 - 3.04) | 0.83  (0.42 - 2.27) |
| Arginase 1 (ng/ml) | 0.66  (0.43 - 1.09) | 0.65  (0.43 - 1.10) | 0.70  (0.39 - 1.10) |
| Arginase 2 (ng/ml) | 15.6  (8.70 - 20.1) | 13.9  (8.70 - 19.2) | 10.2  (6.63 - 14.1) |
| BNP (pg/ml) | 0.15  (0.10 - 0.22) | 0.14  (0.08 - 0.19) | 0.09  (0.06 - 0.14) |
| CCL23 (ng/ml) | 0.22  (0.17 - 0.33) | 0.23  (0.17 - 0.33) | 0.28  (0.21 - 0.35) |
| CRP (μg/ml) | 17.9  (8.98 - 33.0) | 15.7  (7.53 - 32.5) | 12.6  (6.52 - 23.3) |
| CPA-4 (ng/ml) | 2.41  (1.84 - 2.80) | 2.45  (1.85 - 2.99) | 2.82  (2.15 - 3.51) |
| Caspase (ng/ml) | 4.02  (1.90 - 8.48) | 4.08  (1.81 - 8.29) | 3.10  (1.45 - 6.08) |
| CXCL10 (ng/ml) | 0.23  (0.15 - 0.33) | 0.23  (0.16 - 0.32) | 0.22  (0.16 - 0.30) |
| Cystatin C (ng/ml) | 3175  (2234 - 5271) | 3158  (2230 - 5309) | 2789  (1873 - 3880) |
| C-Met 109a (ng/ml) | 123  (92.7 - 147) | 126  (94.2 - 152) | 136  (103 - 171) |
| C-Met 111a (ng/ml) | 356  (291 - 426) | 373  (294 - 441) | 398  (341 - 494) |
| Elafin 131 (ng/ml) | 146  (98.1 - 191) | 133  (96.0 - 180) | 128  (87.8 - 165) |
| Elafin 132 (ng/ml) | 59.18  (41.13 - 121.35) | 61.40  (40.54 - 112.69) | 65.51  (44.99 - 111.07) |
| Endoglin (ng/ml) | 134  (67.5 - 243) | 126  (55.5 - 216) | 33.6  (16.5 - 104) |
| ESAM-1 (ng/ml) | 4.97  (3.99 - 6.11) | 4.97  (4.18 - 6.15) | 5.29  (4.49 - 6.37) |
| Endothelin-1 (pg/ml) | 1.42  (0.91 - 2.40) | 1.42  (0.87 - 2.40) | 1.61  (1.04 - 2.53) |
| Ephrin (pg/ml) * | 94.8% below limit of detection | 95.6% below limit of detection | 97.2% below limit of detection |
| HbF (ng/ml) | 50.9  (26.0 - 90.9) | 50.7  (25.6 - 88.7) | 46.0  (23.1 - 72.7) |
| FIH (ng/ml) | 0.18  (0.08 - 0.41) | 0.19  (0.07 - 0.40) | 0.13  (0.06 - 0.30) |
| ICAM-1 (ng/ml) | 679  (538 - 932) | 665  (517 - 914) | 609  (478 - 828) |
| IL1RA (pg/ml) | 19.7  (13.4 - 30.8) | 19.0  (13.1 - 31.1) | 23.8  (16.6 - 34.5) |
| ST2 116 (ng/ml) | 1.43  (0.76 - 2.43) | 1.21  (0.68 - 2.18) | 0.78  (0.53 - 1.70) |
| ST2 75b (ng/ml) | 6.74  (4.81 - 12.4) | 6.39  (4.49 - 10.2) | 5.86  (3.17 - 9.15) |
| Kunitz-2 (HAI-2) 34a (ng/ml) | 0.42  (0.30 - 0.55) | 0.42  (0.30 - 0.54) | 0.41  (0.29 - 0.56) |
| Kunitz-2 (HAI-2) 35b (ng/ml) | 0.25  (0.12 - 0.39) | 0.26  (0.12 - 0.40) | 0.29  (0.15 - 0.46) |
| Kunitz-2 (HAI-2) 40b (ng/ml) | 0.14  (0.14 - 0.31) | 0.15  (0.14 - 0.31) | 0.24  (0.14 - 0.46) |
| Leptin (ng/ml) | 17.5  (14.1 - 22.9) | 17.2  (13.7 - 22.7) | 17.3  (10.3 - 25.8) |
| Leptin receptor (ng/ml) | 138  (104 - 178) | 140  (105 - 182) | 154  (113 - 199) |
| MIF 49a (ng/ml) | 10.7  (8.97 - 13.1) | 10.5  (8.96 - 13.1) | 11.3  (9.76 - 12.9) |
| MIF 49b (ng/ml) | 8.95  (5.73 - 14.0) | 8.78  (5.72 - 13.7) | 7.88  (5.31 - 11.6) |
| MMP-9 (ng/ml) | 40.3  (30.3 - 54.6) | 39.7  (29.5 - 55.7) | 41.6  (31.5 - 58.4) |
| TIMP-1 (ng/ml) | 132  (94.9 - 187) | 126  (94.7 - 180) | 110  (83.6 - 155) |
| Nephrin 100a (ng/ml) * | 72.9% below limit of detection | 74.6% below limit of detection | 84.7% below limit of detection |
| Nephrin 101a (ng/ml) | 0.42  (0.26 - 0.73) | 0.38  (0.26 - 0.66) | 0.30  (0.16 - 0.49) |
| NGAL (ng/ml) | 48.24  (35.0 - 75.6) | 46.2  (34.2 - 71.0) | 38.7  (24.4 - 56.1) |
| PAPP-A (ng/ml) | 90.7  (40.6 - 154) | 90.0  (40.6 - 156) | 135  (67.4 - 224) |
| Pentraxin 3 64a (ng/ml) * | 77.1% below limit of detection | 77.7% below limit of detection | 72.9% below limit of detection |
| Pentraxin 3 67a (ng/ml) | 3.32  (1.71 - 5.10) | 2.97  (1.68 - 5.03) | 1.97  (0.90 - 3.35) |
| Periostin (ng/ml) | 9.16  (6.86 - 11.3) | 9.07  (6.78 - 11.1) | 8.53  (6.29 - 10.7) |
| PlGF (pg/ml) | 11.6  (5.01 - 33.1) | 16.7  (6.11 - 58.2) | 195  (33.2 - 494) |
| PAI-1 (ng/ml) | 0.50  (0.24 - 0.81) | 0.45  (0.24 - 0.78) | 0.46  (0.26 - 0.73) |
| PAI-2 (ng/ml) | 9.18  (7.39 - 11.9) | 9.93  (7.63 - 12.3) | 11.9  (9.42 - 14.3) |
| Podocalyxin (ng/ml) | 0.16  (0.09 - 0.29) | 0.14  (0.07 - 0.28) | 0.10  (0.07 - 0.19) |
| PCT 95a (pg/ml) | 76.0  (44.6 - 128) | 67.1  (41.2 - 122) | 45.8  (27.5 - 72.1) |
| PCT 99b (pg/ml) | 10.6  (5.63 - 28.9) | 11.5  (5.63 - 28.5) | 11.4  (5.63 - 21.6) |
| ST2 116 (ng/ml) | 1.43  (0.76 - 2.43) | 1.21  (0.68 - 2.18) | 0.78  (0.53 - 1.70) |
| ST2 75b (ng/ml) | 6.74  (4.81 - 12.4) | 6.39  (4.49 - 10.2) | 5.86  (3.17 - 9.15) |
| TGFβ-1 (ng/ml) | 1.77  (1.32 - 2.18) | 1.81  (1.35 - 2.30) | 1.99  (1.62 - 2.43) |
| FasL (ng/ml) * | 72.9% below limit of detection | 74.6% below limit of detection | 68.1% below limit of detection |
| TNFR-1A (ng/ml) | 7.29  (5.01 - 11.6) | 7.67  (5.48 - 11.5) | 7.50  (5.92 - 10.6) |
| FAS (ng/ml) | 2.7  (2.1 - 3.5) | 2.71  (2.10 - 3.53) | 2.72  (2.01 - 3.66) |
| VEGF-C (ng/ml) | 15.2  (12.9 - 18.3) | 15.1  (12.9 - 18.0) | 14.1  (12.1 - 16.6) |
| sFlt-1 (ng/ml) | 3.60  (1.54 - 5.82) | 2.74  (1.33 - 5.38) | 0.95  (0.50 - 2.49) |
| Visfatin (ng/ml) | 2.37  (1.62 - 3.32) | 2.35  (1.61 - 3.29) | 1.93  (1.22 - 3.16) |
| WAP4C HE4 85b (ng/ml) | 1.86  (1.15 - 2.65) | 1.65  (1.12 - 2.63) | 1.40  (0.94 - 1.90) |
| WAP4C HE4 91a (ng/ml) | 13.8  (12.0 - 17.1) | 13.9  (12.2 - 16.8) | 13.3  (11.1 - 15.7) |

* Meaningful quartiles cannot be calculated as the concentrations in most samples were below the lower limit of assay detection.

**Table S9: Test performance statistics (with 95% confidence intervals) for individual indicators and in combination to predict small-for-gestational age (SGA) <10th customized birth-weight centile in 129 women presenting before 35 weeks’ gestation**

| Indicator | Sensitivity % (95% CI) | Specificity % (95% CI) | Positive predictive value % (95% CI) | Negative predictive value % (95% CI) |
| --- | --- | --- | --- | --- |
| AC or EFW <10th centile‡ | 55.8  (44.1 - 67.2) | 92.3  (81.5 - 97.9) | 91.5  (79.6 - 97.6) | 58.5  (47.1 - 69.3) |
| Oligohydramnios § | 15.6  (8.3 - 25.6) | 100  (93.2 - 100) | 100  (73.5 - 100) | 44.4  (35.3 - 53.9) |
| AREDF \|\| | 15.6  (8.3 - 25.6) | 98.1  (89.7 - 100) | 92.3  (64.0 - 99.8) | 44.0  (34.8 - 53.5) |
| PlGF <100 pg/ml | 84.4  (74.4 - 91.7) | 51.9  (37.6 - 66.0) | 72.2  (61.8 - 81.1) | 69.2  (52.4 - 83.0) |
| **Combinations** |  |  |  |  |
| AC or EFW <10th centile or oligohydramnios or AREDF | 58.4  (46.6 - 69.6) | 92.3  (81.5 - 97.9) | 91.8  (80.4 - 97.7) | 60.0  (48.4 - 70.8) |
| AC or EFW <10th centile or PlGF <100 pg/ml | 88.3  (79.0 - 94.5) | 50.0  (35.8 - 64.2) | 72.3  (62.2 - 81.1) | 74.3  (56.7 - 87.5) |

‡ Abdominal circumference or Estimated fetal weight

§ Oligohydramnios defined as amniotic fluid index <5th centile for gestational age

|| Absent or reversed end diastolic flow in umbilical artery Doppler

**Table S10: Test performance statistics (with 95% confidence intervals) for individual indicators and in combination to predict small-for-gestational age (SGA) <3^rd^ customized birth-weight centile in 109 women presenting before 35 weeks’ gestation (excluding those with known abnormal scan findings on day of enrolment)**

| **Indicator** | **Sensitivity %**  **(95% CI)** | **Specificity %**  **(95% CI)** | **Positive predictive value % (95% CI)** | **Negative predictive value % (95% CI)** |
| --- | --- | --- | --- | --- |
| AC or EFW <10th centile ‡ | 61.4  (45.5 - 82.2) | 95.4  (87.1 - 95.4) | 90.0  (73.5 - 97.9) | 78.5  (67.8 - 86.9) |
| Oligohydramnios § | 15.9  (6.6 - 30.1) | 98.5  (91.7 - 100) | 87.5  (47.3 - 99.7) | 63.4  (53.2 - 72.7) |
| AREDF \|\| | 18.2  (8.2 - 32.7) | 98.5  (91.7 – 100.0) | 88.9  (51.8 - 99.7) | 64.0  (53.8 - 73.4) |
| PlGF <100 pg/ml | 93.2  (81.3 - 98.6) | 52.3  (39.5 - 64.9) | 56.9  (44.7 - 68.6) | 91.9  (78.1 - 98.3) |
| **Combinations** |  |  |  |  |
| AC or EFW <10^th^ centile or oligohydramnios or AREDF | 63.6  (47.8 - 77.6) | 93.8  (85.0 - 98.3) | 87.5  (71.0 - 96.5) | 79.2  (68.5 - 87.6) |
| AC or EFW <10th centile or PlGF <100 pg/ml | 95.5  (84.5 - 99.4) | 49.2  (36.6 - 61.9) | 56.0  (44.1 - 67.5) | 94.1  (80.3 - 99.3) |

‡ Abdominal Circumference or Estimated Fetal Weight

§ Oligohydramnios defined as amniotic fluid index <5^th^ centile for gestational age

|| Absent or Reversed End Diastolic Flow in umbilical artery Doppler flow velocity waveforms

**Table S11: Test performance statistics (with 95% confidence intervals) for individual indicators and in combination to predict adverse perinatal outcome in 109 women presenting before 35 weeks’ gestation (excluding those with known abnormal scan findings on day of enrolment)**

| **Indicator** | **Sensitivity %**  **(95% CI)** | **Specificity %**  **(95% CI)** | **Positive predictive value % (95% CI)** | **Negative predictive value % (95% CI)** |
| --- | --- | --- | --- | --- |
| AC or EFW <10th centile ‡ | 39.4  (22.9 – 57.9) | 77.6  (66.6 - 86.4) | 43.3  (25.5 – 62.6) | 74.7  (63.6 - 83.8) |
| Oligohydramnios § | 12.1  (3.4 - 28.2) | 94.7  (87.1 - 98.5) | 50.0  (15.7 - 84.3) | 71.3  (61.4 - 79.9) |
| AREDF \|\| | 12.1  (3.4 - 28.2) | 93.4  (85.3 - 97.8) | 44.4  (13.7 - 78.8) | 71.0  (61.1 -79.6) |
| PlGF <100 pg/ml | 90.9  (75.7 - 98.1) | 44.7  (33.3 - 56.6) | 41.7  (30.2 - 53.9) | 91.9  (78.1 - 98.3) |
| **Combinations** |  |  |  |  |
| AC or EFW <10^th^ centile or oligohydramnios or AREDF | 45.5  (28.1 - 63.6) | 77.6  (66.6 - 86.4) | 46.9  (29.1 -65.6) | 76.6  (65.6 - 85.5) |
| AC or EFW <10th centile or PlGF <100 pg/ml | 93.9  (79.8 -99.3) | 42.1  (30.9 - 54.0) | 41.3  (30.1 - 53.3) | 94.1  (80.3 - 99.3) |

‡ Abdominal Circumference or Estimated Fetal Weight

§ Oligohydramnios defined as amniotic fluid index <5^th^ centile for gestational age

|| Absent or Reversed End Diastolic Flow in umbilical artery Doppler

**Table S12: Characteristics of participants recruited between 35+0 to 36+6 weeks’ gestation at booking and enrolment (grouped by subsequent infant birth weight). Values given are median (quartiles) or n (%) as appropriate.**

| Characteristics | Women with SGA infant <3rd centile  (n = 25) | Women with SGA infant <10th centile (n=39) | Women with infant ≥ 10th centile (n=84) |
| --- | --- | --- | --- |
| At booking:  Age (years) | 32.8 (25.2 - 36.1) | 29.6 (25.1 - 35.3) | 32.4 (28.0 - 35.0) |
| BMI (kg/m2) | 28.6 (25.6 - 30.5) | 28.0 (24.3 - 30.5) | 28.8 (24.3 - 32.9) |
| White ethnicity | 16 (64) | 23 (59) | 53 (63) |
| Highest first trimester systolic BP (mmHg) | 110 (104 - 122) | 116 (104 - 122) | 120 (110 - 128) |
| Highest first trimester diastolic BP (mmHg) | 70 (60 - 75) | 70 (62 - 78) | 76 (67 - 80) |
| Smoker at booking | 6 (24) | 8 (21) | 11 (14) |
| Quit smoking during pregnancy | 4 (16) | 4 (10) | 8 (10) |
| Previous preeclampsia requiring delivery <34/40 | 3 (12) | 4 (10) | 2 (2) |
| Chronic hypertension | 2 (8) | 3 (7) | 6 (7) |
|  |  |  |  |
| At enrolment: |  |  |  |
| Gestational age at sampling (weeks) | 35.7 (35.4 - 36.3) | 36 (35.4 - 36.4) | 36.1 (35.4 - 36.4) |
| New onset hypertension | 20 (80) | 31 (79.5) | 51 (61) |
| Worsening of underlying hypertension | 1 (4) | 3 (7) | 15 (18) |
| New onset of dipstick proteinuria | 18 (72) | 28 (72) | 49 (58) |
| Suspected small for gestational age (customised birth weight centiles) | 8 (32) | 8 (21) | 1 (1) |
| Highest systolic BP (mmHg) | 140 (130 - 151) | 145 (131 - 153) | 143 (132 - 152) |
| Highest diastolic BP (mmHg) | 90 (82 - 98) | 92 (82 - 99) | 94 (87 - 99) |

**Table S13: Characteristics of delivery and maternal and neonatal outcome for women recruited between 35+0 and 36+6 weeks’ gestation. Values given are median (quartiles) or n (%) as appropriate.**

| Characteristics | Women with SGA infant <3rd centile  (n = 25) | Women with SGA infant <10th centile (n=39) | Women with infant ≥ 10th centile (n=84) |
| --- | --- | --- | --- |
| Onset of labour |  |  |  |
| Spontaneous | 4 (16) | 6 (15) | 17 (20) |
| Induced | 16 (64) | 23 (59) | 47 (56) |
| Pre labour caesarean section | 5 (20) | 10 (26) | 19 (23) |
| Mode of delivery |  |  |  |
| Spontaneous | 9 (36) | 13 (33) | 37 (44) |
| Assisted vaginal delivery | 3 (12) | 4 (10) | 7 (8) |
| Emergency caesarean section | 12 (48) | 21 (54) | 40 (48) |
| Adverse maternal outcome* | 6 (24) | 11 (28) | 26 (31) |
| Gestation at delivery (weeks) | 37.3 (36.7 - 37.9) | 37.1 (36.4 - 37.9) | 37.7 (37 - 39.4) |
| Fetal death | 0 (0) | 0 (0) | 0 (0) |
| Neonatal death | 0 (0) | 0 (0) | 0 (0) |
| Birth weight (g) | 2170  (2030 - 2340) | 2250  (2055 - 2480) | 3240  (2925 - 3525) |
| Small for gestational age (<10th birth weight centile) | 25 (100) | 39 (100) | 0 (0) |
| Small for gestational age (<3rd birth weight centile) | 25 (100) | 25 (64.1) | 0 (0) |
| Small for gestational age (<1st birth weight centile) | 11 (44) | 11 (28.2) | 0 (0) |
| Adverse perinatal outcome† | 3 (12) | 4 (10.3) | 5 (6) |

* Adverse maternal outcome defined as presence of any of the following complications: maternal death, eclampsia, stroke, cortical blindness or retinal detachment, hypertensive encephalopathy, systolic blood pressure ≥160mmHg, myocardial infarction, Intubation (other than for caesarean section), pulmonary oedema, platelets <50×10⁹/L (without transfusion), disseminated intravascular coagulation, thrombotic thrombocytopenic purpura/ haemolytic uremic syndrome, hepatic dysfunction (alanine transaminase ≥70IU/L), hepatic haematoma or rupture, acute fatty liver of pregnancy, creatinine >150 μmol/L, renal dialysis, placental abruption, major postpartum haemorrhage, major infection.

†Adverse perinatal outcome defined as: presence of any of the following complications: Antepartum/ intrapartum fetal or neonatal death, Neonatal unit admission for >48 hrs at term, Intraventricular haemorrhage, Periventricular leukomalacia, seizure, retinopathy of prematurity, respiratory distress syndrome, bronchopulmonary dysplasia or necrotising enterocolitis.

**Table S14: Individual biomarker ROC areas (with 95% confidence intervals) when sampled between 35+0 and 36+6 weeks’ gestation.**

|  | Women with SGA infant <3^rd^ centile | Women with SGA infant <10^th^ centile |
| --- | --- | --- |
| ADAM-9 | 0.55 (0.49 - 0.62) | 0.56 (0.50 - 0.62) |
| Angiogenin | 0.61 (0.49 - 0.73) | 0.57 (0.46 - 0.68) |
| ANP | 0.49 (0.35 - 0.63) | 0.54 (0.42 - 0.65) |
| Arginase 1 | 0.61 (0.48 - 0.73) | 0.54 (0.42 - 0.65) |
| Arginase 2 | 0.67 (0.55 - 0.79) | 0.64 (0.54 - 0.75) |
| BNP | 0.65 (0.54 - 0.77) | 0.68 (0.58 - 0.78) |
| CCL23 | 0.52 (0.40 - 0.65) | 0.54 (0.43 - 0.65) |
| CRP | 0.68 (0.57 - 0.80) | 0.59 (0.48 - 0.70) |
| CPA-4 | 0.72 (0.61 - 0.83) | 0.72 (0.63 - 0.82) |
| Caspase | 0.58 (0.46 - 0.70) | 0.52 (0.42 - 0.63) |
| CXCL10 | 0.46 (0.32 - 0.61) | 0.51 (0.40 - 0.63) |
| Cystatin C | 0.53 (0.39 - 0.66) | 0.54 (0.44 - 0.65) |
| C-Met 109a | 0.64 (0.51 - 0.76) | 0.64 (0.54 - 0.74) |
| C-Met 111a | 0.64 (0.52 - 0.76) | 0.64 (0.54 - 0.74) |
| Elafin 131 | 0.64 (0.52 - 0.76) | 0.55 (0.44 - 0.66) |
| Elafin 132 | 0.60 (0.49 - 0.72) | 0.52 (0.42 - 0.63) |
| Endoglin | 0.58 (0.46 - 0.70) | 0.65 (0.55 - 0.76) |
| ESAM-1 | 0.67 (0.55 - 0.79) | 0.66 (0.55 - 0.76) |
| Endothelin-1 | 0.57 (0.45 - 0.70) | 0.55 (0.45 - 0.66) |
| Ephrin | 0.51 (0.50 - 0.52) | 0.51 (0.49 - 0.52) |
| HbF | 0.55 (0.41 - 0.69) | 0.49 (0.38 - 0.61) |
| FAS | 0.53 (0.40 - 0.65) | 0.55 (0.45 - 0.66) |
| FasL | 0.52 (0.41 - 0.62) | 0.54 (0.45 - 0.62) |
| FIH | 0.56 (0.43 - 0.68) | 0.51 (0.40 - 0.62) |
| ICAM-1 | 0.67 (0.56 - 0.78) | 0.60 (0.49 - 0.71) |
| IL-1ra | 0.62 (0.50 - 0.75) | 0.62 (0.51 - 0.73) |
| Kunitz-2 (HAI-2) 34a | 0.36 (0.24 - 0.48) | 0.40 (0.30 - 0.51) |
| Kunitz-2 (HAI-2) 35b | 0.74 (0.63 - 0.85) | 0.73 (0.65 - 0.82) |
| Kunitz-2 (HAI-2) 40b | 0.69 (0.57 - 0.81) | 0.68 (0.58 - 0.78) |
| Leptin | 0.50 (0.37 - 0.63) | 0.49 (0.38 - 0.60) |
| Leptin receptor | 0.65 (0.52 - 0.78) | 0.62 (0.51 - 0.73) |
| MIF -49a | 0.57 (0.45 - 0.70) | 0.57 (0.46 - 0.68) |
| MIF- 49b | 0.48 (0.36 - 0.60) | 0.49 (0.38 - 0.59) |
| MMP-9 | 0.57 (0.45 - 0.69) | 0.56 (0.45 - 0.67) |
| TIMP-1 | 0.56 (0.45 - 0.68) | 0.52 (0.41 - 0.63) |
| Nephrin 100a | 0.53 (0.44 - 0.62) | 0.52 (0.45 - 0.59) |
| Nephrin 101a | 0.67 (0.55 - 0.80) | 0.63 (0.52 - 0.74) |
| NGAL | 0.46 (0.33 - 0.58) | 0.49 (0.39 - 0.60) |
| PAPP-A | 0.62 (0.50 - 0.74) | 0.66 (0.56 - 0.77) |
| Pentraxin- 3 64a | 0.46 (0.36 - 0.57) | 0.45 (0.36 - 0.54) |
| Pentraxin- 3 67a | 0.59 (0.46 - 0.71) | 0.61 (0.50 - 0.71) |
| Periostin | 0.50 (0.37 - 0.63) | 0.57 (0.46 - 0.68) |
| PAI-1 | 0.49 (0.36 - 0.62) | 0.48 (0.37 - 0.59) |
| PAI-2 | 0.67 (0.56 - 0.79) | 0.65 (0.55 - 0.75) |
| PlGF | 0.69 (0.57 - 0.81) | 0.74 (0.64 - 0.83) |
| Podocalyxin | 0.65 (0.53 - 0.76) | 0.62 (0.52 - 0.72) |
| PCT 95a | 0.61 (0.48 - 0.73) | 0.59 (0.48 - 0.69) |
| PCT 99b | 0.38 (0.26 - 0.51) | 0.45 (0.34 - 0.55) |
| sFlt-1 | 0.57 (0.45 - 0.70) | 0.61 (0.51 - 0.71) |
| ST2-116b | 0.55 (0.43 - 0.67) | 0.61 (0.51 - 0.72) |
| ST2 -75b | 0.49 (0.35 - 0.62) | 0.55 (0.44 - 0.66) |
| TGFβ-1 | 0.66 (0.55 - 0.77) | 0.64 (0.54 - 0.74) |
| TNFR-1A | 0.60 (0.47 - 0.73) | 0.57 (0.47 - 0.68) |
| VEGF-C | 0.42 (0.29 - 0.56) | 0.46 (0.35 - 0.58) |
| Visfatin | 0.46 (0.35 - 0.57) | 0.49 (0.39 - 0.60) |
| WAP4C HE4 85b | 0.52 (0.39 - 0.66) | 0.55 (0.45 - 0.66) |
| WAP4C HE4 91a | 0.40 (0.27 - 0.53) | 0.47 (0.35 - 0.58) |

**Table S15: ROC areas (with 95% confidence intervals) for individual biomarkers and combinations (derived from logistic regression) to predict small for gestational age (SGA) <3rd and <10th customized birth-weight centiles in women presenting between 35+0 and 36+6 weeks’ gestation. [ ] low concentrations of biomarker/ ratio correlated to severe disease.**

| Biomarkers or combinations | Women with SGA infant <3rd centile | Women with SGA infant <10^th^ centile |
| --- | --- | --- |
| Nephrin | 0.67 (0.55 - 0.80) | 0.63 (0.52 - 0.74) |
| [PlGF] | 0.69 (0.57 - 0.81) | 0.74 (0.64 - 0.83) |
| [CPA-4] | 0.72 (0.61 - 0.83) | 0.72 (0.63 - 0.82) |
| Kunitz-2 (HAI-2) | 0.74 (0.63 - 0.85) | 0.73 (0.65 - 0.82) |
| **Combinations** |  |  |
| [PlGF/s-Flt ratio] | 0.66 (0.54 - 0.78) | 0.70 (0.60 - 0.80) |
| [PlGF/Endoglin ratio] | 0.66 (0.54 - 0.78) | 0.73 (0.63 - 0.82) |
| [PlGF], Nephrin | 0.73 (0.62 - 0.84) | 0.76 (0.67 - 0.85) |
| [PlGF], [CPA-4] | 0.77 (0.67 - 0.88) | 0.81 (0.73 - 0.90) |
| [PlGF], Nephrin, [CPA-4] | 0.77 (0.66 - 0.88) | 0.81 (0.73 - 0.90) |

**Table S16: Test performance statistics for individual indicators and in combination to predict small-for-gestational age (SGA) <3^rd^ customized birth-weight centile in 53 women presenting between 35+0 and 36+6 weeks’ gestation**

| Indicator | Sensitivity % (95% CI) | Specificity % (95% CI) | Positive predictive value % (95% CI) | Negative predictive value % (95% CI) |
| --- | --- | --- | --- | --- |
| AC or EFW <10th centile‡ | 64.3  (35.1 - 87.2) | 94.9  (82.7 - 99.4) | 81.8  (48.2 - 97.7) | 88.1  (74.4 - 96.0) |
| Oligohydramnios§ | 14.3  (1.8 - 42.8) | 94.9  (82.7 - 99.4) | 50.0  (6.8 - 93.2) | 75.5  (61.1 - 86.7) |
| AREDF \|\| | 14.3  (1.8 - 42.8) | 97.4  (86.5 - 99.9) | 66.7  (9.4 - 99.2) | 76.0  (61.8 - 86.9) |
| PlGF <100 pg/ml | 85.7  (57.2 - 98.2) | 23.1  (11.1 - 39.3) | 28.6  (15.7 - 44.6) | 81.8  (48.2 - 97.7) |
| Combinations |  |  |  |  |
| AC or EFW <10th centile or oligohydramnios or AREDF | 71.4  (41.9 - 91.6) | 89.7  (75.8 - 97.1) | 71.4  (41.9 - 91.6) | 89.7  (75.8 - 97.1) |
| AC or EFW <10th centile or PlGF <100 pg/ml | 92.9  (66.1 - 99.8) | 23.1  (11.1 - 39.3) | 30.2  (17.2 - 46.1) | 90.0  (55.5 - 99.7) |

‡ Abdominal circumference or Estimated fetal weight

§ Oligohydramnios defined as amniotic fluid index <5th centile for gestational age

|| Absent or reversed end diastolic flow in umbilical artery Doppler

**Table S17: Test performance statistics for individual indicators and in combination to predict small-for-gestational age (SGA) <10^th^ customized birth weight-centile in 53 women presenting between 35+0 and 36+6 weeks’ gestation**

| Indicator | Sensitivity % (95% CI) | Specificity % (95% CI) | Positive predictive value % (95% CI) | Negative predictive value % (95% CI) |
| --- | --- | --- | --- | --- |
| AC or EFW <10th centile‡ | 52.4  (29.8 - 74.3) | 100  (89.1 - 100) | 100  (71.5 - 100) | 76.2  (60.5 - 87.9) |
| Oligohydramnios§ | 14.3  (3.0 - 36.3) | 96.9  (83.8 - 99.9) | 75.0  (19.4 - 99.4) | 63.3  (48.3 - 76.6) |
| AREDF \|\| | 9.5  (1.2 - 30.4) | 96.9  (83.8 - 99.9) | 66.7  (9.4 - 99.2) | 62.0  (47.2 - 75.3) |
| PlGF <100 pg/ml | 90.5  (69.6 - 98.8) | 28.1  (13.7 - 46.7) | 45.2  (29.8 - 61.3) | 81.8  (48.2 - 97.7) |
| Combinations |  |  |  |  |
| AC or EFW <10th centile or oligohydramnios or AREDF | 57.1  (34.0 - 78.2) | 93.8  (79.2 - 99.2) | 85.7  (57.2 - 98.2) | 76.9  (60.7 - 88.9) |
| AC or EFW <10th centile or PlGF <100 pg/ml | 95.2  (76.2 - 99.9) | 28.1  (13.7 - 46.7) | 46.5  (31.2 - 62.3) | 90.0  (55.5 - 99.7) |

‡ Abdominal circumference or Estimated fetal weight

§ Oligohydramnios defined as amniotic fluid index <5th centile for gestational age

|| Absent or reversed end diastolic flow in umbilical artery Doppler

**Table S18: Test performance statistics for individual indicators and in combination to predict adverse perinatal outcome in 53 women presenting between 35+0 and 36+6 weeks’ gestation**

| Indicator | Sensitivity % (95% CI) | Specificity % (95% CI) | Positive predictive value % (95% CI) | Negative predictive value % (95% CI) |
| --- | --- | --- | --- | --- |
| AC or EFW <10th centile‡ | 50.0  (6.8 - 93.2) | 81.6  (68.0 - 91.2) | 18.2  (2.3 - 51.8) | 95.2  (83.8 - 99.4) |
| Oligohydramnios§ | 0  (0 - 60.2) | 91.8  (80.4 - 97.7) | 0  (0 - 60.2) | 91.8  (80.4 - 97.7) |
| AREDF \|\| | 25.0  (0.6 - 80.6) | 95.9  (86.0 - 99.5) | 33.3  (0.8 - 90.6) | 94.0  (83.5 - 98.7) |
| PlGF <100 pg/ml | 100  (39.8 - 100) | 22.4  (11.8 - 36.6) | 9.5  (2.7 - 22.6) | 100  (71.5 - 100) |
| Combinations |  |  |  |  |
| AC or EFW <10th centile or oligohydramnios or AREDF | 50.0  (6.8 - 93.2) | 75.5  (61.1 - 86.7) | 14.3  (1.8 - 42.8) | 94.9  (82.7 - 99.4) |
| AC or EFW <10th centile or PlGF <100 pg/ml | 100  (39.8 - 100) | 20.4  (10.2 - 34.3) | 9.3  (2.6 - 22.1) | 100  (69.2 - 100) |

‡ Abdominal circumference or Estimated fetal weight

§ Oligohydramnios defined as amniotic fluid index <5th centile for gestational age

|| Absent or reversed end diastolic flow in umbilical artery Doppler
